# MaxDIA enables library-based and library-free data-independent acquisition proteomics

**DOI:** 10.1038/s41587-021-00968-7

**Published:** 2021-07-08

**Authors:** Pavel Sinitcyn, Hamid Hamzeiy, Favio Salinas Soto, Daniel Itzhak, Frank McCarthy, Christoph Wichmann, Martin Steger, Uli Ohmayer, Ute Distler, Stephanie Kaspar-Schoenefeld, Nikita Prianichnikov, Şule Yılmaz, Jan Daniel Rudolph, Stefan Tenzer, Yasset Perez-Riverol, Nagarjuna Nagaraj, Sean J. Humphrey, Jürgen Cox

**Affiliations:** 1 Computational Systems Biochemistry Research Group, Max-Planck Institute of Biochemistry, Martinsried, Germany; 2 Chan Zuckerberg Biohub, San Francisco, CA USA; 3 Evotec München GmbH, Martinsried, Germany; 4 Institute for Immunology, Johannes Gutenberg University, Mainz, Germany; 5 Bruker Daltonik, GmbH, Bremen, Germany; 6 Bosch Center for Artificial Intelligence, Renningen, Germany; 7 European Molecular Biology Laboratory, European Bioinformatics Institute (EMBL-EBI), Wellcome Trust Genome Campus, Hinxton, Cambridge, UK; 8 School of Life and Environmental Sciences, Charles Perkins Centre, University of Sydney, Camperdown, New South Wales Australia; 9 Department of Biological and Medical Psychology, University of Bergen, Bergen, Norway

**Keywords:** Proteome informatics, Proteomics

## Abstract

MaxDIA is a software platform for analyzing data-independent acquisition (DIA) proteomics data within the MaxQuant software environment. Using spectral libraries, MaxDIA achieves deep proteome coverage with substantially better coefficients of variation in protein quantification than other software. MaxDIA is equipped with accurate false discovery rate (FDR) estimates on both library-to-DIA match and protein levels, including when using whole-proteome predicted spectral libraries. This is the foundation of discovery DIA—hypothesis-free analysis of DIA samples without library and with reliable FDR control. MaxDIA performs three- or four-dimensional feature detection of fragment data, and scoring of matches is augmented by machine learning on the features of an identification. MaxDIA’s bootstrap DIA workflow performs multiple rounds of matching with increasing quality of recalibration and stringency of matching to the library. Combining MaxDIA with two new technologies—BoxCar acquisition and trapped ion mobility spectrometry—both lead to deep and accurate proteome quantification.

## Abstract (web summary)

The software platform MaxDIA streamlines analysis of data-independent acquisition proteomics.

## Main

DIA proteomics^1^ promises robust and accurate quantification of proteins over large-scale study designs and across heterogeneous laboratory conditions^2^. In all omics sciences, robust data analysis pipelines are as important as the data acquisition technology itself, and proteomics is no exception. MaxQuant^3–6^ is the most widely used software for analyzing data-dependent acquisition (DDA) proteomics data, providing a vendor-neutral complete end-to-end solution for all common experimental designs. With version 2.0, described here, MaxQuant offers an equally complete DIA software infrastructure, termed MaxDIA. Such a unified framework over all mass spectrometry-based proteomics based on peptide quantification comes with several advantages over existing software^7–10^. DDA libraries and DIA samples can be processed in integrated, consistent ways. Algorithmic parts of the workflow that do not depend on the type of acquisition, like protein quantification algorithms (such as MaxLFQ^11^), protein redundancy grouping or protein-level FDR, can be applied to all data in exactly the same way, making DDA and DIA studies much more comparable.

The classical approach to DIA data analysis uses a spectral library of peptides, which are queried in the DIA samples and quantified in case of their presence. In this spectral library-based approach, the rate of false matches can, in principle, be controlled with techniques similar to those developed in DDA proteomics^12^. For instance, the target-decoy method^13^ has been adapted to DIA^9^. Additionally, several library-free approaches exist^14^, and spectral predictions have been successfully used for DIA data analysis^15–20^. However, effective control of FDRs, in particular on the level of identified proteins with library-free methods, although having been attempted by other software^9,10^, is still a critical aspect that requires thorough investigation. In case reliability of library-free identifications is achieved, DIA can additionally be employed in a discovery mode, without biases imposed by a library and, at the same time, with certainty that the identified set of proteins contains, at most, a predefined percentage of false positives—for example, 1%, as is standardly applied in DDA-based proteomics. Here we demonstrate that MaxDIA fulfills these criteria and can, indeed, be used in such a discovery DIA mode.

Machine learning is an integral part of MaxDIA. We use the bi-directional recurrent neural network^21^ (BRNN) approach termed DeepMass:Prism^15^ to create, in silico, very precise libraries of tandem mass spectrometry (MS/MS) spectra for peptides digested from complete proteome sequence databases. BRNNs are also used for the dataset-specific prediction of liquid chromatography retention times. Furthermore, to score library DIA sample matches based on multivariate information derived from properties of the matches, we apply the gradient boosting method XGBoost^22^, which is highly superior to using only the matching score itself and also compared to applying other machine learning approaches.

High-quality three-dimensional (3D) or, in the presence of ion mobility data, 4D feature detection^3,23^ of the precursor data is one of the components of MaxQuant for DDA data, leading to noise suppression. In MaxDIA, fragment ions are additionally detected as 3D/4D features. Besides noise removal, this ensures that data are not over-interpreted. The feature detection on fragment data allows to require that all signals belonging to a 3D/4D peak contribute as evidence to only one peptide identification, ensuring that signals at slightly different retention times or ion mobility values, but really belonging to the same feature, are not used as independent evidence for two similar peptides—for example, differing by a modification or resulting from an amino acid polymorphism.

In MaxDIA, we support two new and promising technologies, both of which enable deep quantification of DIA samples. One is to combine DIA with high-dynamic-range precursor data obtained by the BoxCar acquisition method^24^. The second is to use ion mobility as an extra data dimension on a trapped ion mobility spectrometry quadrupole time of flight (timsTOF Pro) mass spectrometer^25–27^ for DIA. Both increase the quantified proteome in DIA samples, substantially providing highly precise and linear quantification over the whole dynamic range. Furthermore, because the MaxLFQ algorithm was designed to perform label-free quantification on pre-fractionated samples^11^, MaxDIA also has the capability to perform label-free quantification of pre-fractionated samples analyzed by DIA, which opens up applications of DIA requiring ultra-deep proteome quantification. Complete submissions to the PRoteomics IDEntifications^28^ (PRIDE) database using an adapted mzTab^29^ scheme can also be performed automatically using MaxDIA.

## Results

### MaxDIA data analysis workflow

**Fig. 1:**
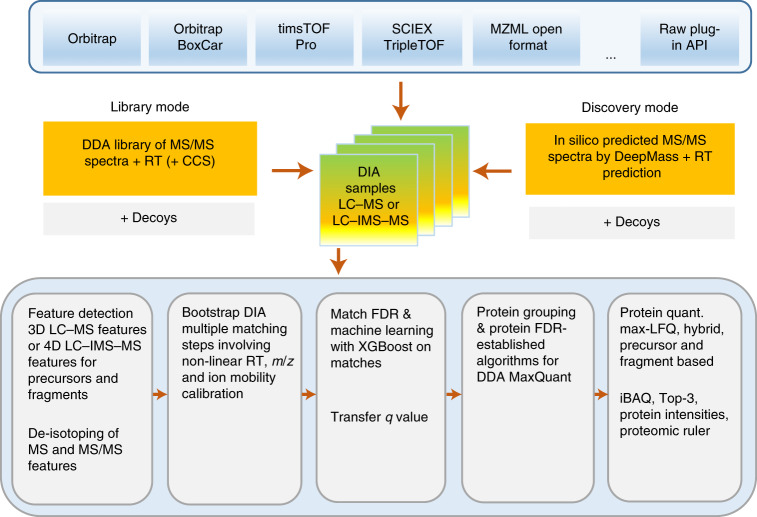
Overview of the MaxDIA workflow. MaxDIA can be operated in library and discovery mode. Many concepts and algorithms—for instance, for protein quantification—are re-used from the conventional MaxQuant workflow for DDA data and have been further developed for DIA. This results in an end-to-end DIA software that contains many established MaxQuant concepts, such as label-free quantification with MaxLFQ or iBAQ quantification. RT, retention time.

**Fig. 2:**
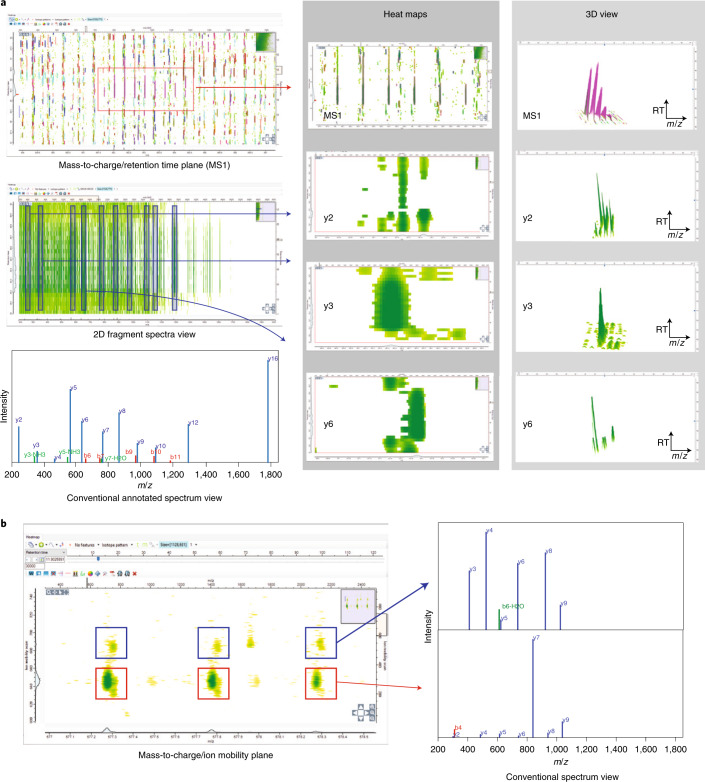
3D/4D feature detection of precursors and fragments. **a**, Visualization of precursors and fragments of a peptide measured on an Orbitrap. The raw data can be visualized together with the peak detection results as heat maps and 3D models for precursor and fragment data in the graphical user interface of MaxQuant. **b**, Two peptides with nearly equal mass, both with charge 2 and having very similar retention times, are resolved by ion mobility on a timsTOF Pro mass spectrometer. A heat map visualizes intensities as a function of retention time and collision cross-section for the precursor isotope patterns. The two respective MS/MS spectra of fragments assigned to the precursors are shown. RT, retention time.

MaxDIA is embedded into the MaxQuant software environment (Fig. 1) and shares with it the graphical user interface, computational infrastructure and many algorithmic workflow components applicable to both. It is vendor neutral, with direct support for the most common native vendor file formats for reading mass spectra, as well as the open mzML file format^30^. Generic DIA acquisition modes are supported, including overlapping windows, variable window sizes, pooled multiple windows and variable *m*/*z*–ion mobility regions for timsTOF instruments. MaxDIA can be operated in a classical library-based approach or in discovery DIA mode. In the former, DIA datasets are interrogated within MaxQuant by spectral libraries generated with MaxQuant, whereas the latter does not require acquisition of a spectral library. In discovery DIA mode, spectral libraries are generated by DeepMass:Prism^15^, a BRNN that enables precise prediction of spectral intensities from peptide sequences. Decoy spectra are generated by reverting library sequences under the constraint of preserving the cleavage characteristics of the protease that was used in the experiment and ensuring that the decoy peptide masses, retention times and ion mobility values follow the same multivariate distribution as the target peptides. DIA samples and libraries are then analyzed in an end-to-end workflow for peptide and protein identification and quantification. MaxQuant’s 3D or 4D feature detection^3,23^ (Fig. 2) and de-isotoping are performed on the precursor data and on all liquid chromatography with tandem mass spectrometry (LC–MS/MS) or LC–ion mobility spectrometry (IMS)–MS/MS fragment data domains corresponding to precursor selection windows. Defining MS/MS features in a multi-dimensional way is particularly important for fragment data, because it avoids over-interpretation of identification results. This enables the requirement that every MS/MS feature is used at most once in peptide identification. Problems might arise if such precautions are not taken, because features will be double-counted for the identification of peptides that are similar to each other due to sequence homology or due to the presence or absence of a modification but for which there is insufficient evidence for the existence of both peptide forms.

### Bootstrap DIA

Central to the workflow is bootstrap DIA, which consists of multiple steps of matching the library spectra to DIA samples (Supplementary Fig. 1). These steps aim to bootstrap the DIA identification process based on the least possible prior knowledge. Bootstrap DIA replaces and substantially extends the concept of the ‘first search–main search’ strategy^31^ as well as the ‘retention time alignment’ and ‘match between runs’ used in DDA MaxQuant. Increasingly more information is gained in each round, with this information used in subsequent rounds. For instance, in the first round of matching, no retention time constraint is used. Based on these matches, a linear model is fit between the library and sample retention times, which is used to align runs to one another, even when gradient lengths substantially differ. This linear correction can be applied to the data, and, in the second round of matching, retention times can be filtered based on a time window that is automatically adapted to the distribution of all retention time differences after linear alignment. This filtering removes sufficiently many false-positive matches, so that, from the third round of matching, a non-linear retention time recalibration function can be determined. Application of the non-linear recalibration function allows to subsequently apply more stringent filtering. Similar multi-step recalibration and filtering steps are applied to precursor and fragment masses as well as to collision cross-sections, if applicable. Supplementary Fig. 2 shows how target decoy distributions are affected after each matching step with increasingly more stringent filers. The resulting non-linear precursor and fragment *m*/*z* recalibrations depending on *m*/*z* and retention time are shown in Supplementary Figs. 3 and 4.

A consequence of the bootstrap DIA process is that precursor and fragment masses, retention times and ion mobility values are non-linearly aligned between each DIA sample and library without the need for spike-in standards. A prerequisite for this is that the DDA runs in the datasets used for the library are well aligned to each other, because the precision of alignment between library and DIA samples is otherwise limited by the variability of retention times and collision cross-sections within the library. Therefore, when processing libraries in MaxQuant, retention time and ion mobility alignments should be activated. A challenging attribute that can be learned from the data is non-linear retention time mappings between library and samples. This means that gradients between library and DIA runs do not need to be the same, and label-free quantification is possible even between DIA measurements with different gradient lengths. To evaluate the matching of different DIA gradient durations to a library, we generated a DDA library consisting of 16 high-pH reversed-phase fractions of a HeLa cell lysate measured with 25-min gradients and measured the same sample unfractionated with DIA using 30-, 60-, 90- and 120-min gradients. Supplementary Fig. 5 shows retention time alignments between the library and DIA samples, and precise quantification among samples with different gradient lengths is shown in Supplementary Fig. 6. These capabilities greatly enhance the flexibility of MaxDIA, making the software applicable to analyzing a broader range of samples.

### Scoring of library-to-sample matches by machine learning

To quantify the quality of match between a library spectrum and a DIA sample at a given retention time and collisional cross-section (CCS) value, if applicable, we first find a precursor feature and all fragment features that match to the library spectrum with tolerances for *m*/*z*, retention time and CCS, dependent on the matching step in the bootstrap DIA workflow. To measure the match quality, we then calculate a score, which is the sum over all matching features of numbers between 0 and 1, each quantifying how far away from the apex the respective peak was hit (Supplementary Fig. 7). For a given library spectrum, this score is maximized over retention time and ion mobility. It is then ensured, through a second round of scoring, that every feature in a DIA sample is used, at most, for one library spectrum match.

This score then is enhanced through machine learning. To this end, we construct a feature space that, in addition to the score, contains various properties of the match (Supplementary Fig. 8), such as mass errors (in p.p.m.) for precursor and fragment ions as deviations from the theoretical masses calculated from elemental compositions. Also, the errors of retention times and ion mobilities are included in the feature space. An interesting feature is the apex fraction, which is the ratio of the intensity at the current retention time to the maximum peak intensity. We employ a classification algorithm to separate ‘target’ from ‘decoy’ hits based on this feature space. We define the machine learning-based match score as the assignment probability to the ‘target’ class of the machine learning algorithm. This is a number expressing the affinity to the ‘target’ spectra as opposed to the ‘decoy’ spectra. To eliminate the risk of overfitting, we determine these machine learning scores in five-fold cross-validation, such that a match for which the machine learning score is calculated has not been used for training the model that is used for its prediction.

We used several different classification algorithms and monitored their effect on the identification performance of MaxDIA. We compared the performances of XGBoost^22^, fully connected multi-hidden layer neural networks, random forests^32^ and AdaBoost (Supplementary Fig. 9), scanning, for each algorithm, suitable ranges of meta-parameters. We found that XGBoost performs best among the tested algorithms, in contrast to Demichev et al.^10^, who found neural networks to perform favorably. This choice is also different from DDA where, for similar purposes, support vector machine-based methods are used^33^. XGBoost provides information on the importance of features for classification (Supplementary Fig. 8). We found that, in the library-based approach, the feature defining whether the precursor has an isotope pattern assigned or was seen only as a single peak is of greater importance than the raw score itself. Furthermore, retention time, precursor mass errors, number of modifications and missed cleavages were among the top ten highest ranked features. Also among the top ten is the ‘sample fragment overlap’, which quantifies if and to what extent the N- and C-terminal ion series are overlapping in the DIA sample, thereby placing restrictions on the precursor mass.

### Identification performance and quantification precision

**Fig. 3:**
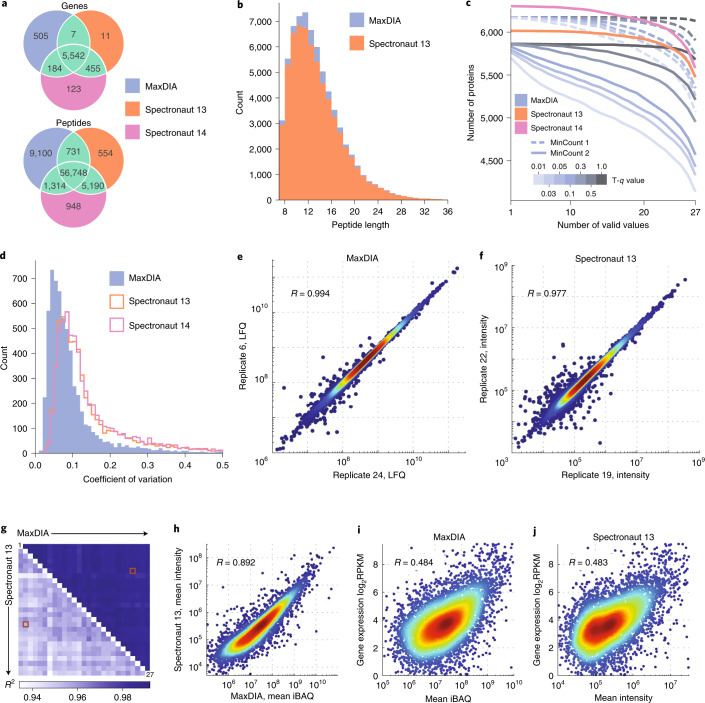
Performance evaluation. Twenty-seven technical replicates of HepG2 cell lysate were analyzed on an Orbitrap mass spectrometer (Methods). **a**, Number of identified protein groups with 1% FDR on protein and peptide level and number of peptides at 1% library-to-DIA-sample FDR obtained with MaxDIA, Spectronaut 13 and Spectronaut 14. **b**, Histograms of peptide lengths identified with MaxDIA (blue) and Spectronaut 13 (red). **c**, Number of proteins with, at most, *x* out of 27 valid values for Spectronaut 13 (red), Spectronaut 14 (magenta) and MaxDIA with MaxLFQ minimum ratio count = 1 (blue, dashed) and = 2 (blue, solid). Multiple curves for the two MaxQuant series of curves correspond to seven different choices for the transfer *q* value (0.01, 0.03, 0.05, 0.1, 0.3, 0.5 and 1). **d**, Histograms of coefficients of variation for analyses with default settings in MaxDIA (solid blue) and in Spectronaut 13 and Spectronaut 14 (open). **e**, log–log scatter plot of LFQ intensities between two representative replicates obtained with MaxQuant. The two replicates were chosen to have the median Pearson correlation of all pairwise replicate comparisons. **f**, Same as in **e** for Spectronaut intensities. Similarly, the two replicates were chosen to represent the median Pearson correlation coefficient of all pairwise comparisons. **g**, Heat map with all pairwise Pearson correlations among the 27 replicates for MaxDIA (upper triangle) and Spectronaut (lower traingle). The two values corresponding to the comparisons in **e** and **f** are marked with red squares. **h**, log–log scatterplot of iBAQ protein intensities from MaxDIA against Spectronaut protein intsnsities. **i**, log–log scatterplot of MaxDIA iBAQ values averaged over the replicates against RPKM values from RNA-seq data. **j**, Same as **i** with protein intensities from Spectronaut.

To evaluate the performance of MaxDIA, we ran it, as well as Spectronaut 13 and Spectronaut 14, on a dataset comprising 27 technical replicate injections of peptides derived from the human HepG2 cell line measured in DIA as well as a DDA library created from 12 high-pH reversed-phase fractions (Methods). Using default parameters in both software, including a 1% FDR on precursor and protein levels, we obtained 6,238 protein groups mapped to Entrez Gene identifiers with MaxDIA compared to 6,015 with Spectronaut 13 and 6,304 with Spectronaut 14, with an overlap of 5,542 among all software platforms (Fig. 3a). MaxDIA found 7.4% more peptides than Spectronaut 13 and 5.8% more than Spectronaut 14 at 1% library-to-DIA-matches FDR. We found several peptide properties to be similarly distributed among the identification results of the two software platforms (Supplementary Fig. 10), including retention time, precursor charge and mass-to-charge ratio and precursor mass error. In addition, the length distribution of identified peptides was very similar between the two analysis software packages (Fig. 3b). Peptides that were uniquely found by MaxQuant were biased toward low signal intensity (Supplementary Fig 10a).

Although DIA is thought to be better in terms of data completeness^34,35^ compared to DDA, we observe that this depends on the algorithmic details, and that there is a tradeoff between data completeness and confidence of protein identification within a specific sample, as opposed to the whole dataset. After identifying peptides and proteins for the whole dataset, we apply a ‘transfer *q-*value’ cutoff to the identifications of matches in each sample. Setting it to 1 implies that no sample-specific restrictions are applied and that the peptide is quantified, whenever any evidence is found for its existence. A transfer *q* value of 0.01 (equal to the global *q* value of library-to-sample matches) results in stringent identification in every sample and, hence, certainty about the actual sample-specific presence of peptides and proteins. We scanned through seven values of the transfer *q* value between 0.01 and 1 and monitored the number of proteins that have a certain number or fewer valid values in terms of label-free quantification (LFQ) intensities (Fig. 3c). As expected, for larger transfer *q* values, the curves are flatter and higher in terms of total protein numbers. When using 1 for the ‘minimum ratio count’ parameter of the LFQ algorithm, most parts of all curves are above the line for the Spectronaut 13 software and slightly below for the Spectronaut 14 software. For ‘minimum ratio count’ = 2, which ensures higher accuracy of quantification, the array of curves is intersecting with the Spectronaut curves. The ‘minimum ratio count’ parameter requires at least that many peptide features to be shared for a protein in a specific comparison between two samples^11^. After evaluating the accuracy of benchmark quantification results on several mass spectrometry platforms (see, for instance, Supplementary Fig. 15 for timsTOF data), we decided to select 0.3 as the default value for the transfer *q* value. Study-specific objectives (completeness of quantification versus certainty of identification in individual samples) might suggest deviations from this default value.

The distribution of coefficients of variation (CVs) (Fig. 3d) indicates substantially higher quantification precision obtained with MaxLFQ (described below) in MaxDIA compared to both Spectronaut versions, with median CVs of 0.072, 0.109 and 0.114, respectively. Figure 3e,f shows typical log–log scatter plots of protein intensities between replicates displaying fewer outliers and higher Pearson correlation for MaxDIA. All pairwise replicate Pearson correlations of logarithmic intensities are represented as a heat map in Fig. 3g for both programs, showing consistently higher correlations for MaxDIA (median 0.993) compared to Spectronaut (median 0.977). We found a good overall agreement between averaged Spectronaut intensities and MaxDIA intensity-based absolute quantification (iBAQ) values (Fig. 3h) with a Pearson correlation of 0.87. We performed mRNA versus protein copy number comparisons based on reads per kilobase per million mapped reads (RPKM)^36^ and iBAQ^37^ values, respectively, using MaxDIA and Spectronaut (Fig. 3i,j). Both comparisons showed similar correlations between mRNA and protein levels, which are also compatible with correlations typically found in such studies^38^.

### Accuracy of FDR estimates and discovery DIA

**Fig. 4:**
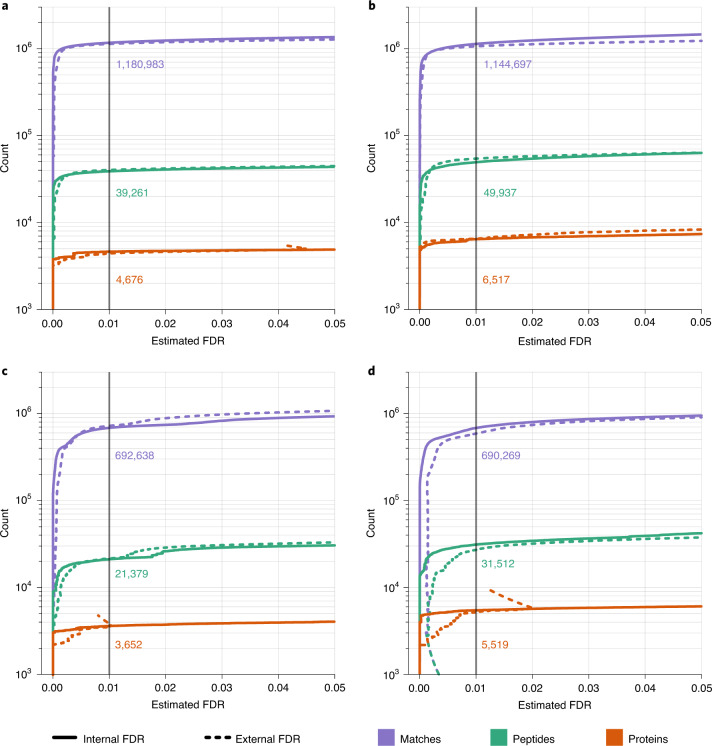
Internal and external FDR. **a**, Number of identifications (blue: matches; green: peptides; red: protein groups) as a function of estimated FDR. The FDR is estimated once with the ‘internal’ target-decoy method implemented in MaxQuant (solid lines) and once with the ‘external’ method using mixing maize and human samples for generating the library and using only human sample in the DIA runs (dashed lines). **b**, Same as in **a** but using in silico predicted libraries generated using DeepMass:Prism^15^ **c**, Same as **a** but using the raw score instead of the machine learning–derived score. **d**, Same as **b** but using the raw score instead of the machine learning–derived score.

To evaluate the reliability of FDR estimates using MaxDIA’s target-decoy strategy, we used a pooled DDA library generated from mixed human and maize samples, with corresponding DIA runs comprising only human samples^34^. Hence, every match identified as being derived from the maize proteome is a known false-positive identification (having discarded peptides that are shared among proteins of the two species). This enables calculation of an ‘external’ FDR, which is calculated independently of the ‘internal’ FDR estimated by the decoy approach in MaxDIA. Figure 4a compares internal and external FDRs on match, peptide and protein group levels. The curves for internal and external FDR are in very good agreement on all three levels. When comparing the numbers of identified matches, peptides and protein groups at 1% FDR, which is often taken as a default value in shotgun proteomics, the numbers differed by only 3.0%, 3.4% and 5.0%, respectively, between internally and externally controlled FDRs. Hence, our decoy-based FDR estimates are in good agreement with external FDR calculations.

Given these results, we investigated how accurate the FDR estimates are for cases in which the library is dissimilar to the DIA sample. Hence, we assembled a library of in silico predicted spectra based on DeepMass:Prism^15^ consisting of all tryptic peptides digested from all human UniProt^39^ sequences (Release 2019_05 containing 20,959 proteins) without missed cleavages. We additionally generated predicted retention times for each in silico spectrum based on a BRNN used previously for the same purpose^15^. Using this library with the same DIA dataset as in Fig. 4a, we generated the same curves for internal and external FDRs as before (Fig. 4b). Here as well, we observed good agreement between internal and external FDRs. In particular, at an FDR of 1%, the number of identified protein groups differed by only 1.5%. We did, however, identify 39% more protein groups with the in silico library compared to the measured library. This highlights that MaxDIA does not require that spectral libraries are generated from matching samples in a project-specific manner, and yet FDRs are still reliably controlled. This enables the use of MaxDIA in a ‘discovery’ mode (discovery DIA), which is not biased by a library and completely hypothesis free in terms of which proteins can be found, by using in silico predicted libraries for all protein sequences. We repeated all analysis while replacing the DeepMass:Prism algorithm with two other spectral prediction methods—wiNNer^15^ and PROSIT^16^—indicating that there are no substantial differences resulting from different choices among these prediction algorithms (Supplementary Fig. 11).

We additionally repeated these analyses using the raw matching score instead of the machine learning-improved score (Fig. 4c,d). This revealed that the agreement of internal and external FDR does not depend on whether the XGBoost-based machine learning was used to adjust the scoring. However, the use of machine learning did substantially increase peptide (83% and 58% for library DIA and discovery DIA, respectively) and protein group (28% and 18%, respectively) identifications.

### MaxLFQ adaptation for DIA

A prime example of the re-use and continued development of algorithms from DDA MaxQuant to MaxDIA is the label-free quantification algorithm MaxLFQ^11^. Here, quantification is based on first calculating all pairwise peptide ratios between samples, which are then summarized by the intensity profile that best fits all the pairwise ratios. This procedure can be generalized to DIA by replacing a single ratio per peptide with multiple ratios derived from precursor intensities and from the most intense fragment peaks (Supplementary Fig. 12). This approach naturally implements hybrid quantification of precursor and fragment intensities.

**Fig. 5:**
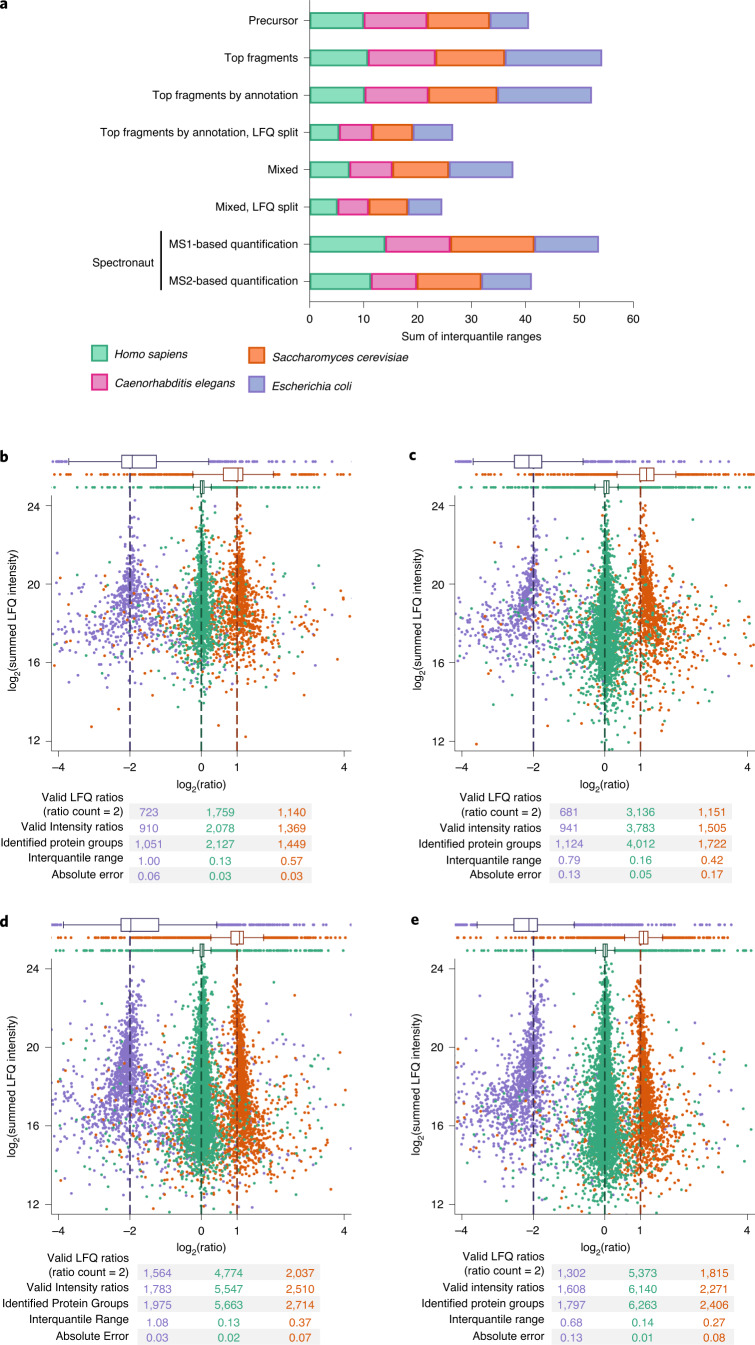
MaxLFQ for DIA. **a**, Stacked interquartile rages of protein ratio distributions in the small-ratio four-species dataset from Bruderer et al.^34^ using different versions of MaxLFQ for DIA and compared to the results from this publication. MaxDIA is capable of MS1 and MS2 level as well as hybrid quantification modes. **b**, Quantification of a three-species benchmark mixture measured on a SCIEX TripleTOF 6600 instrument mixing proteomes from three species in defined ratio^2^ with MaxLFQ for DIA. The accompanying DDA library was used. The box plots here and in the subsequent panels are based on the numbers of data points given in the tables below the respective plot (valid LFQ ratios). All box plots indicate the median and the first and third quartiles as box ends. Whiskers are positioned 1.5 box lengths away from the box ends. **c**, Same as **b** but analyzed with MaxDIA in discovery mode. **d**, Quantification of a three-species benchmark mixture measured on a Bruker timsTOF Pro instrument mixing proteomes from three species in defined ratio using a DDA library. **e**, Same as **d** but analyzed in discovery mode.

To benchmark quantification accuracy, we downloaded a four-species dataset with well-defined small ratios between replicate groups^34^. Ratios are expected to be 0%, 10%, 20% or 30%, depending on the species comprising: *Homo sapiens*, *Caenorhabditis elegans*, *Saccharomyces cerevisiae* and *Escherichia coli*. We tested several combinations of precursor, fragment or mixed quantification and fragment intensities summed up or kept separately. We measured the variability as the interquartile range of ratios within each species and summed these over the four species (Fig. 5a). We found that hybrid quantification between precursors and fragments with fragment intensities kept separate for individual ion types in LFQ resulted in the smallest quantification errors measured as the sum of the interquartile ranges of ratio distributions over the four species. The accuracy observed exceeded both MS1- and MS2-level quantification reported by Bruderer et al.^34^. A further question is how the filtering of fragments by their intensity improves quantification accuracy. To this end, we used only the top N intense peaks for quantification while varying N (Supplementary Fig. 13a). We found that accuracy increases with the number of fragments used, indicating that no filtering of fragments by intensity is required. Similarly, we investigated whether filtering to the top N most intense peptides per protein is beneficial (Supplementary Fig. 13b), finding that it is best to use all available peptides.

In recent years, several researchers have worked on approaches to remove interferences and improve the selection of transitions in DIA analysis^40–43^. Although this approach to improving quantification has its merits, in this study we followed a different strategy with MaxLFQ to obtain high accuracy on the level of protein groups. Single-fragment features that are interfered by overlapping features and, due to this, have incorrect intensities will not affect protein quantification in MaxDIA much because the protein-level quantification relies solely on the medians of peptide signal ratios (Supplementary Fig. 12c). Hence, even if a fraction of signals is affected by interferences, they are expected to drop out in the calculation of the median over multiple fragments and peptides. We compared MaxLFQ in MaxDIA to Avant-garde curated Skyline quantification on a multi-species benchmark dataset simulating realistic biological data^41^. We found that the transition-filtered quantification provided by Avant-garde is not systematically better than the MaxLFQ quantification in MaxDIA (Supplementary Fig. 14).

Next, we analyzed a quantitative benchmark dataset obtained on a SCIEX TripleTOF 6600 instrument, mixing proteomes from three species in defined ratios among replicate groups^2^ (Fig. 5b). Using the original library analyzed with MaxQuant and using default values for all parameters, we identified 4,627 protein groups and achieved linear quantification for all three species over the whole dynamic range. In discovery mode with a predicted library allowing for one missed tryptic cleavage, the number of identified protein groups rose by 48% to 6,858 (Fig. 5c), with, on average, improved quantification accuracy for the species with ratios as measured by interquartile ranges of species-specific ratio distributions. *H. sapiens,* which expresses a much larger number of proteins, received the largest increase, identifying almost two-fold more protein groups (4,012 versus 2,127), whereas *C. elegans* and *E. coli* received proportionally fewer additional proteins.

We next acquired a quantitative three-species benchmark dataset using ion mobility on a Bruker timsTOF Pro instrument. Using the DDA library acquired on the same instrument type, we identified 10,352 protein groups. We again used MaxLFQ for DIA with hybrid quantification with separate intensities for each fragment ion (Fig. 5d), seeing excellent quantification over the whole dynamic range without non-linearities. In discovery mode (Fig. 5e), the number of identified protein groups increases to 10,466 with higher quantification accuracy, again judged by the interquartile ranges of ratio distributions. Scanning through the transfer *q* value, we found that quantification accuracy was best with a value near 0.3 (Supplementary Fig. 15).

### BoxCar and fractionated DIA

**Fig. 6:**
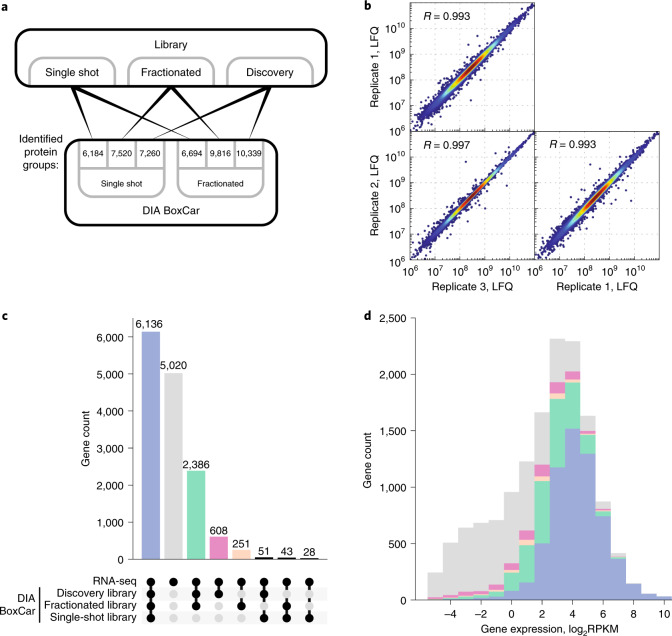
BoxCar and fractionated DIA. **a**, Schedule of libraries and DIA samples. Three different library approaches—single-shot, deep-fractionated and discovery mode—were compared to single-shot, deep-fractionated DIA samples. **b**, MaxLFQ quantification among three replicates of fractionated BoxCar DIA samples analyzed in discovery DIA mode. All pairwise Pearson correlations are above 0.99. **c**, Venn diagram-like comparison represented as bar plot between RNA-seq data of HEK cells and three different library methods applied to the fractionated DIA samples. All data have been mapped to gene identifiers **d**, Histogram of protein identifications mapped to gene identifiers sorted into bins according to log_2_ RPKM values of the RNA-seq data.

We recently implemented analysis of data acquired using the BoxCar acquisition method in MaxQuant in the DDA context^24^, whose primary goal is to achieve higher dynamic range for the precursor intensities. Because this should be beneficial for DIA as well, we implemented its generalization to combining high-dynamic-range precursor measurements with DIA acquisition for the fragments. Furthermore, it is possible with MaxDIA to analyze and quantify DIA samples that have been pre-fractionated on peptide or protein levels. This feature can be applied to all supported instruments and DIA acquisition methods. To highlight these features, we acquired both DDA libraries and DIA measurements from HEK cell lysate as single shots and as high-pH reversed-phase peptide fractionated samples, which were pooled into eight fractions for MS analysis (Methods). We analyzed all combinations of libraries and samples, and, in addition, we analyzed the DIA samples in discovery DIA mode allowing for one missed trypsin cleavage (Fig. 6a). For the fractionated DIA samples, we observed an increase in the number of identified protein groups concomitant with the size of the library, with the most identifications in discovery mode. With single-shot samples, the number of identified proteins saturates with library size, having slightly more identifications with the fractionated library. However, comparing identifications for the single-shot DIA samples between fractionated library and discovery mode, we found that the results were very similar, with 89% overlap of Entrez Gene identifier mapped protein groups (Supplementary Fig. 16). For a comparison of protein identifications for different fractionation depths of the DIA samples, see Supplementary Fig. 17. This indicates that, for both types of DIA samples, it is not compulsory to produce a deep, fractionated library, but that similar, or even better, results can be achieved in discovery DIA mode. Quantification with MaxLFQ among three replicates of fractionated DIA samples showed very good correlation, with a median Pearson correlation of 0.993 (Fig. 6b).

We then compared the results obtained with the three different library creation approaches to RNA sequencing (RNA-seq) data of HEK cells (Methods). Figure 6c compares the four sets of identifications based on gene identifiers. Of the 9,503 genes covered by proteomics methods, 65% were found with all three library methods. An additional 25% were found with both discovery mode and fractionated library but not with the single-shot library. In total, 608 proteins were uniquely found with the discovery approach, compared to 251 with the deep-fractionated library, suggesting preference for the discovery mode from the perspective of results, in addition to its economic advantages. In Fig. 6d, the results from Fig. 6c are displayed according to RPKM intervals of the RNA-seq data. The RNA-seq data show a bimodal left shoulder that is typical of expression noise^44^, genes for which there is only limited proteomic evidence of translation. As expected, highly abundant proteins are recovered with all methods, whereas, at low abundance, both the deep-fractionated library and discovery DIA approach add identifications.

## Discussion

Here we introduce MaxDIA, a complete end-to-end DIA workflow embedded into the MaxQuant environment with major new features and broad applicability to established and novel MS technologies. We demonstrate the widespread and general utility of the software, including its use in analyzing BoxCar DIA and ion mobility DIA data, demonstrating very high proteome quantification coverage.

This framework lends itself to several extensions that are currently under development. In particular, although the analysis of post-translational modifications (PTMs) is possible, in principle, by providing suitable libraries with spectra from modified peptides, proper localization of the modification on the peptide has to be carefully implemented as an additional process after peptide identification^45^. For these purposes, a PTM score guiding localization needs to be calculated directly from the DIA data and not from extracted spectra. Similarly, extensions to the identification of cross-linked peptides are straightforward^46^ and are planned for future releases of MaxDIA.

## Methods

### HepG2 technical replicate data

#### Cell culture and MS sample preparation

HepG2 cells were from the American Type Culture Collection and cultured in MEM and 10% FCS. Cells were washed twice with ice-cold PBS and harvested using freshly prepared SDC buffer (1% SDC, 10 mM TCEP, 40 mM CAA, 75 mM Tris-HCl pH 8.5). The SDC lysates were heated to 95 °C for 10 min while shaking at 750 r.p.m. in a ThermoMixer (Eppendorf) and then sonicated for 10 min (10 × 30-s on/off cycles) using a Bioruptor Pico sonication device (Diagenode). Protein concentrations were determined using the 660-nm assay (Thermo Fisher Scientific), and the proteins were digested with trypsin/Lys-C mix (Promega, V5071) overnight at 37 °C with a 1:50 enzyme-to-protein ratio. The digestion was stopped by adding 2 volumes of 99% ethylacetate/1% trifluoroacetic acid (TFA), followed by sonication for 1 min using an ultrasonic probe device (energy output ~40%). The samples were then de-salted using in-house-prepared, 200-µl, two-plug SDB-RPS StageTips^47^ (3M Empore, 2241). SDB-RPS StageTips were conditioned with 60 µl of isopropanol, 60 µl of 80% ACN/5% NH_4_OH and 100 µl of 0.2% TFA. The SDC/ethylacetate mixture was directly loaded onto the tips, followed by two washing steps of 200 µl of 0.2% TFA each. Peptides were eluted with 80% ACN/5% NH_4_OH, speedvac dried and then resupended in 0.1% formic acid (FA). After estimation of the concentration using a NanoDrop device (Thermo Fisher Scientific), the samples were adjusted to 0.4 µg µl^−1^ with 0.1% FA, of which 2 µl (800 ng) was injected into the mass spectrometer.

#### LC–MS/MS measurements

Peptides were loaded on 40-cm reversed-phase columns (75-µm inner diameter, packed in-house with ReproSil-Pur C18-AQ 1.9-µm resin (ReproSil-Pur, Dr. Maisch)). The column temperature was maintained at 60 °C using a column oven. An EASY-nLC 1200 system (Thermo Fisher Scientific) was directly coupled online with the mass spectrometer (Q Exactive HF-X, Thermo Fisher Scientific) via a nano-electrospray source, and peptides were separated with a binary buffer system of buffer A (0.1% FA plus 5% DMSO) and buffer B (80% acetonitrile plus 0.1% FA plus 5% DMSO) at a flow rate of 250 nl min^−1^. The mass spectrometer was operated in positive polarity mode with a capillary temperature of 275 °C. The samples were acquired with a DIA method established by Bruderer et al.^34^. Briefly, the method consisted of an MS1 scan (*m*/*z,* 300–1,650) with an AGC target of 3 × 10^6^ and a maximum injection time of 60 ms (*R* = 120,000). DIA scans were acquired at *R* = 30,000, with an AGC target of 3 × 10^6^, ‘auto’ for injection time and a default charge state of 4. The spectra were recorded in profile mode, and the stepped collision energy was 10% at 25%.

#### High-pH reversed-phase fractionation

HepG2 cells were lysed as described in ‘Cell culture and MS sample preparation’. Next, 150 µg of total protein was digested with a trypsin/Lys-C mix (Promega, V5071) overnight at 37 °C with a 1:50 enzyme-to-protein ratio. The digestion was stopped by adding 2 volumes of 99% ethylacetate/1% TFA, followed by sonication for 1 min using an ultrasonic probe device (energy output ~40%). The peptides were de-salted using 30-mg (8B-S029-TAK) Strata-X-C cartridges (Phenomenex) as follows: (1) conditioning with 1 ml of isopropanol; (2) conditioning with 1 ml of 80% ACN/5% NH_4_OH; (3) equilibration with 1 ml of 99% ethylacetate/1% TFA; (4) loading of the sample; (5) washing with 2 × 1 ml of 99% ethylacetate/1% TFA; (6) washing with 1 ml of 0.2% TFA; and (7) elution with 2 × 1 ml of 80% ACN/5% NH_4_OH. The eluates were snap-frozen in liquid nitrogen and lyophilized overnight. The lyophilized peptides were resuspended in 400 µl of 0.1% FA and fractionated using a 3 × 250-mm XBridge column (Waters) on an ÄKTA HPLC system (GE Healthcare). Fractionation was performed with a flow rate of 0.5 ml min^−1^ and with a constant flow of 10% 25 mM ammonium bicarbonate, pH 10. Peptides were separated using a linear gradient of ACN from 7% to 30% over 15 min, followed by a 5-min increase to 55% ACN and a subsequent ramping to 100% ACN. Fractions were collected at 50-s intervals in 15-ml Falcon tubes to a total of 36 fractions and then pooled to obtain 12 fractions (A1-B1-C1, A2-B2-C2, etc.). All fractions were acidified by addition of FA to a final amount of 0.1% and then lyophilized. Peptides were subsequently resuspended in 100 µl of 0.1% TFA and de-salted using in-house-prepared C18 STAGE tips^47^ as follows: (1) equilibration with 100 µl of isopropanol; (2) equilibration with 100 µl of 0.1% TFA; (3) loading of the sample; (4) washing with 100 µl pf 0.1% FA; and (5) elution with 30 µl of 80% acetonitrile/0.1% FA. Peptides were speedvac dried and resupended in 20 µl of 0.1% FA, and the concentration was estimated on a NanoDrop device (Thermo Fisher Scientific). The samples were then adjusted to 0.4 µg µl^−1^ with 0.1% FA, of which 2 µl (800 ng) was injected into the mass spectrometer.

### HeLa data with varying gradients

#### High-pH reversed-phase peptide fractionation

Next, 6 µg of HeLa peptides were loaded onto a Waters BEH130 C18 2.1 × 250-mm column in 90 µl of MS loading buffer at a flow rate of 0.5 ml min^−1^ using a Dionex Ultimate 3000 HPLC, and column temperature was maintained at 50 °C. After loading, a binary gradient of 10% buffer A (2% acetonitrile, 10 mM ammonium formate, pH 9) to 40% buffer B (80% acetonitrile, 10 mM ammonium formate, pH 9) was formed over 4.4 min, followed by a washout from 40% to 100% buffer B over 1 min, after which the column was held at 100% buffer B for 10 min before re-equilibration. Fractions were collected over a period of 6.4 min from the first peptide elution, with fraction collection each 8 s and automatic concatenation into 16 fractions (200 µl fraction volume). Fractions were dried down in a vacuum concentrator (Eppendorf) and resuspended in MS loading buffer (0.3% TFA, 2% acetonitrile).

#### MS analysis

Peptides were loaded onto a 40-cm column with a 75 µM inner diameter, packed in-house with 1.9 µM C18 ReproSil particles (Dr. Maisch). Column temperature was maintained at 60 °C with a column oven (Sonation). A Dionex UltiMate 3000 RSLCnano HPLC system (Thermo Fisher Scientific) was interfaced with a Q Exactive HF-X benchtop Orbitrap mass spectrometer (Thermo Fisher Scientific) using a Nanospray Flex ion source (Thermo Fisher Scientific). For all samples, peptides were separated with a binary buffer system of 0.1% (vol/vol) FA (buffer A) and 80% (vol/vol) acetonitrile/0.1% (vol/vol) FA (buffer B), and peptides were eluted at a flow rate of 400 nl min^−1^. Gradient ranges and durations were as follows: 5–40% buffer B over 30 min (DDA library); 3–19% buffer B over 10 min and 19–41% over 5 min (15 min DIA gradient); 3–19% buffer B over 20 min and 19–41% over 10 min (30 min DIA gradient); 3–19% buffer B over 40 min and 19–41% over 20 min (1-h DIA gradient); 3–19% buffer B over 60 min and 19–41% over 30 min (1.5-h DIA gradient); and 3–19% buffer B over 80 min and 19–41% over 40 min (2-h DIA gradient). For the DDA library, peptides were analyzed with one full scan (350–1,400 *m*/*z*, *R* = 60,000 at 200 *m*/*z*) with a target of 3 × 10^6^ ions, followed by up to 20 data-dependent MS/MS scans with higher energy collision dissociation (HCD; target 1 × 10^5^ ions, maximum injection time (IT) 28 ms, isolation width 1.4 *m*/*z*, NCE 27%, intensity threshold 3.7 × 10^5^), detected in the Orbitrap (*R* = 15,000 at 200 *m*/*z*). Dynamic exclusion was enabled (15 s). For DIA measurements, peptides were analyzed with one full scan (350–1,400 *m*/*z*, *R* = 120,000 at 200 *m*/*z*) at a target of 3 × 10^6^ ions, followed by 48 data-independent MS/MS scans spanning 350–975 *m*/*z* with HCD (target 3 × 10^6^ ions, maximum IT 22 ms, isolation width 14 *m*/*z*, NCE 25%), detected in the Orbitrap (*R* = 15,000 at 200 *m*/*z*).

### Three-species timsTOF Pro benchmark data

#### Sample preparation

Human cervix carcinoma cell line HeLa was purchased from the German Resource Center for Biological Material. Cells were cultured in Iscove’s Modified Dulbecco Medium (PAN-Biotech) supplemented with 10% (vol/vol) FCS (Thermo Fisher Scientific), 1% (vol/vol) glutamine (Carl Roth) and 1% (vol/vol) sodium pyruvate (Serva) at 37 °C in a 5% CO_2_ environment. A pure culture of the *S. cerevisiae bayanus* strain Lalvin EC-1118 was obtained from the Institut Oenologique de Champagne. Yeast cells were grown in YPD media as described by Fonslow et al.^48^. *E. coli* (TOP10) cells were purchased from Thermo Fisher Scientific and grown in LB liquid medium. After harvesting, cells were lysed by adding a urea-based lysis buffer (7 M urea, 2 M thiourea, 5 mM DTT, 2% (wt/vol) CHAPS). Lysis was promoted by sonication at 4 °C for 15 min using a Bioruptor (Diagenode). After cell lysis, protein amounts were determined using the Pierce 660-nm Protein Assay (Thermo Fisher Scientific) according to the manufacturer’s protocol. Tryptic digestion applying a modified filter-aided sample preparation^49^ protocol was performed as described in detail previously^50^. To generate the two hybrid proteome samples, tryptic peptides were combined in the following ratios as detailed previously^2,50]^. Sample A was composed of 65% wt/wt human, 30% wt/wt yeast and 5% wt/wt *E. coli* proteins. Sample B was composed of 65% wt/wt human, 15% wt/wt yeast and 20% wt/wt *E. coli* proteins.

#### LC–MS analysis

Samples were analyzed by LC–MS on a timsTOF Pro (Bruker Daltonik), which was coupled online to a nanoElute nanoflow liquid chromatography system (Bruker Daltonik) via a CaptiveSpray nano-electrospray ion source. Peptides (corresponding to 200 ng) were separated on a reversed-phase C18 column (25 cm × 75 µm i.d., 1.6 µm, IonOpticks). Mobile phase A was water containing 0.1% (vol/vol) FA, and mobile phase B was acetonitrile containing 0.1% (vol/vol) FA. Peptides were separated running a gradient of 2–37% mobile phase B over 100 min at a constant flow rate of 400 nl min^−1^. Column temperature was controlled at 50 °C. MS analysis of eluting peptides was performed in diaPASEF mode. For diaPASEF, we adapted the instrument firmware to perform data-independent isolation of multiple precursor windows within a single TIMS separation (100 ms). We used a method with two windows in each 100-ms diaPASEF scan. Sixteen of these scans covered the diagonal scan line for doubly charged and triply charged peptides in the *m/z*–ion mobility plane with narrow 25-*m/z* precursor windows, resulting in a total cycle time of 1.6 s.

### BoxCar DIA HEK data

#### Cell culture and MS sample preparation

HEK293 cells were grown in DMEM supplemented with penicillin, streptomycin and 10% FCS. Cells were washed twice with ice-cold PBS before scraping in PBS and centrifugation at 300*g* for 6 mins at 4 °C. Supernatant was aspirated and the pellet lysed in 2.5% SDS buffered with 50 mM Tris pH 8.1 and heated to 95 °C for 5 min, before probe sonication. The BCA assay was used to quantify the protein content of centrifuge-clarified lysates before precipitation with 5 volumes of acetone. Pellets were resuspended in 50 mM Tris pH 8.1 containing 8 M urea, reduced with 1 mM DTT and alkylated with 5 mM IAA before initiation of digestion overnight with LysC at an enzyme-to-protein ratio of 1:100. The digest mixture was diluted four-fold, and trypsin was added at an enzyme-to-protein ratio of 1:100 for 6 h, followed by an additional aliquot of trypsin overnight. Digestion was stopped by acidification to 1% TFA, placed on ice for 5 min and centrifuged to remove insoluble material. Peptides were de-salted with mixed-mode SPE cartridges (Strata-XC, Phenomenex), activated with 100% methanol, conditioned with 80% acetonitrile/0.1% TFA and equilibrated with 0.2% TFA, which was followed by sample loading, washing with 99.9% isopropanol/0.1% TFA, washing twice with 0.2% TFA and washing once with 0.1% FA, before elution with 60% acetonitrile/0.5% ammonium hydroxide. Eluate was flash-frozen and dried by centrifugal evaporation.

#### Offline peptide fractionation

Peptides were resuspended in buffer A (10 mM ammonium bicarbonate) and injected onto a 4.6 × 250-mm 3.5-μm Zorbax 300 Extend-C18 column. Peptides were separated on a non-linear gradient exactly as described (ref. ^51^), using the following composition of buffer B (10 mM ammonium bicarbonate, 90% acetonitrile). Peptide fractions were frozen at −80 °C before centrifugal evaporation. Peptides were resuspended in 1% TFA and concatenated at by combining every 24th fraction for the library or every 8th fraction for the fractionated BoxCar DIA runs, using fractions 13–90.

Concatenated or non-fractionated samples were de-salted with SEP-PAK tC18 SPE cartridges (Waters), activated with 100% methanol, conditioned with 80% acetonitrile/0.1% TFA and equilibrated with 0.2% TFA. After sample loading, cartridges were washed with 0.5, 1 and 3 cartridge volumes of 0.2% TFA and eluted with 1 volume of 80% acetonitrile/0.1% TFA and then frozen before drying in a centrifugal evaporator.

Next, 1 µg of peptide was loaded onto an Aurora 25 cm × 75 µm ID, 1.6-µm C18 column (IonOpticks) maintained at 40 °C. Peptides were separated with an EASY-nLC 1200 system at a flow rate of 300 nl min^−1^ using a binary buffer system of 0.1% FA (buffer A) and 80% acetonitrile with 0.1% FA (buffer B) in a two-step gradient from 3% to 27% B in 105 min and from 27% to 40% B in 15 min. All scans were recorded in the Orbitrap of a Fusion Lumos instrument running Tune version 3.3, equipped with a nanoFlex ESI source, operated at 1.6 kV, and the RF lens was set to 30%. The scan sequence was initiated with MS1 scans from 350 to 1,650 *m*/*z* recorded at 120,000 resolution, with an AGC target of 250% and maximum injection time of 246 ms. The mass range was divided into 24 segments of variable width, with three BoxCar scans (multiplexed targeted SIM scan) isolating eight segments per scan, comprising every third segment. The segments used were identical to those in the MS2 scans, retaining a 1-*m*/z overlap between boxes in adjacent scans. The normalized AGC target was 200% per segment, with a maximum injection time of 246 ms. BoxCar scans were also recorded at a resolution of 120,000. This was followed by 24 MS2 scans from 200 to 2,000 *m*/z with windows as previously described (ref. ^34^). Fragmentation was induced with HCD using stepped collision energy of 22%, 27% and 32% for the window center. Each MS2 scan was recorded at a resolution of 30,000 and an AGC target of 1,000%, with a maximum injection time of 60 ms.

### Data downloads

In addition to the data measured for this publication, we downloaded the following publicly available datasets. The four-species mixture dataset^34^ containing *H. sapiens*, *C. elegans*. *S. cerevisiae* and *E. coli* with ratios of 0%, 10%, 20% and 30%, respectively, among replicate groups was downloaded from ProteomeXchange (PXD005573). SCIEX TripleTOF 6600 three-species benchmark data^2^ were obtained from ProteomeXchange (PXD002952). The HepG2 RNA-seq data are part of the ENCODE dataset^52^ and were downloaded from the Sequence Read Archive (SRA) (SRP014320). The HEK RNA-seq data are part of the Cell Atlas dataset^53^ and were downloaded from the SRA (SRP017465).

### Data analysis

In all MaxQuant analyses for generating libraries and for analyzing DIA samples (MaxDIA), version 2.0.0 was used, and, for all parameters, the default values were used unless stated otherwise. In particular, MaxQuant was run with a transfer *q* value of 0.3 unless stated otherwise. Searches were performed with the following FASTA files from UniProt: UP000005640_9606 (*H. sapiens*), UP000007305_4577 (*Z. mays*), UP000002311_559292 (*S. cerevisiae*), UP000000625_83333 (*E. coli*) and UP000001940 (*C. elegans*). Methionine oxidation and protein N-terminal acetylation were used as variable modifications in all searches, as is default in MaxQuant.

#### Comparing number of proteins among datasets

Proteins are assembled into protein groups for identification to account for the redundancy of protein sequences with regard to the peptide evidence distinguishing them. This works in MaxDIA in exactly the same way as in the standard DDA usage of MaxQuant. These protein groups are dataset dependent, and, hence, comparisons between two protein groups tables—for instance, in Venn diagrams or between a protein groups table and RNA-seq data—are non-trivial. Here, we follow the route of mapping all protein identifiers in a protein group to Entrez Gene identifiers^54^. In the vast majority of cases, protein groups map to single gene identifiers. For cases in which they map to more than one, both gene identifiers are taken into the set. For counting protein group identifications, we always remove protein groups that are flagged as ‘reverse’ or ‘only identified by site’. For human datasets, we removed protein groups denoted as ‘potential contaminant’ only if they are of non-human origin and kept human proteins, which consist mostly of human keratins. For the dataset containing bovine plasma, the proteins in the standard MaxQuant contaminant list of bovine origin were not removed.

#### FDR curves

For estimating external FDR, we used a combination of human and maize libraries from ref. ^34^ or of human and maize predicted libraries in discovery mode on the human HepG2 DIA samples. For analyzing library-to-DIA-sample matches and peptide identifications in Fig. 4, we do not apply a protein-level FDR and scan through the library-to-DIA-sample FDR. It is crucial to take this approach, in particular when comparing numbers of identifications with other software, because, when applying protein-level FDR in MaxQuant, peptides that are not mapping to a protein identified at the specified protein FDR are discarded, unlike in most other software packages. For obtaining the protein-level FDR curves in Fig.4, we applied a library-to-DIA-sample match FDR of 1%. Peptides that are shared between human and maize proteins were discarded. The sizes of the FASTA files were, for *H. sapiens*, 20,962 + additional 75,485 records, resulting in 1,525,028 unique peptide sequences for one trypsin missed cleavage. For *Z. mays*, there were 39,400 + additional 59,878 records, resulting in 1,765,195 unique peptide sequences. We used a correction factor of 1.176 to account for the size differences, which corresponds to the ratio of total amino acid positions in the two databases.

#### RNA-seq data analysis

Raw reads were filtered using trimmomatic^55^ (v0.36) using default parameters for paired-end data. Filtered reads were mapped to the human reference genome GRCh38 (Ensemble release 100) using STAR^56^ aligner (v2.5.3a). Further processing—sorting, converting from SAM to BAM format and indexing—was done using SAMtools^57^ (v1.6). Gene expression quantification (RPKM) for protein-coding genes was performed in Perseus^58^ (v1.6.14.0).

#### Spectronaut analysis

Raw MS data were processed using Spectronaut version 13.10.191212 and Spectronaut version 14.10.201222 using default settings, using a spectral library generated by searching using MaxQuant version 1.6.10.43. To determine the influence on the results of non-default parameter settings, we varied several of them as shown in Supplementary Fig. 10g.

### Software development, requirements, availability and usage

MaxDIA was developed in conjunction with MaxQuant in C#, runs on Windows and Linux operating systems and requires .NET Core 2.1. In addition, .NET Framework 4.7.2 has to be installed on Windows. The graphical user interface version is currently restricted to Windows. A platform-neutral command line version is available. MaxQuant is efficiently running in parallel on arbitrarily many CPUs on single-node platforms. Having 4 GB of memory per parallel running thread is recommended. Disk space should be at least twice the space that is used by the raw data. MaxQuant, including MaxDIA, can be downloaded from https://www.maxquant.org/. MaxDIA is included in the standard MaxQuant release from version 2.0.0 onward. How to use MaxDIA in library or discovery mode is described in the accompanying Supplementary Notes document. It also contains a list of all user-definable parameters with a description of their meaning.

### PRIDE support

We support complete submissions to the PRIDE database^28^ for the DIA identification results. We extended the mzTab format^29^ to cover DIA data sets. For this purpose, new controlled vocabulary terms were introduced, along with additional external reference files. These external reference files contain DIA library matches with mass, intensity and annotation information in a spectral library format (MSP format). MaxQuant will generate a new output folder called ‘combined\msp’ into which these results are written. A user must provide this folder in addition to raw and mzTab files during submission to PRIDE. More details on a complete PRIDE submission are provided in the Supplementary Notes. This is the first instance of complete PRIDE submissions for DIA datasets.

### Reporting Summary

Further information on research design is available in the Nature Research Reporting Summary linked to this article

## Online content

Any methods, additional references, Nature Research reporting summaries, source data, extended data, supplementary information, acknowledgements, peer review information; details of author contributions and competing interests; and statements of data and code availability are available at 10.1038/s41587-021-00968-7.

## Supplementary information

Supplementary Information Supplementary Figs. 1–17 and Notes.

Reporting Summary

## Data Availability

The MS proteomics data have been deposited to the ProteomeXchange Consortium (http://proteomecentral.proteomexchange.org) via the PRIDE partner repository with the dataset identifiers PXD022582 (DDA data) and PXD022589 (DIA data, also containing MaxQuant v2.0.0).

## Code availability

MaxQuant is freeware, and the code is partially open and available at https://github.com/JurgenCox/compbio-base. All custom code used in generating figures is available at https://github.com/cox-labs/DIAtools.
