# Supplementary material for: MaxDIA enables library-based and library-free data-independent acquisition proteomics
Source: Nat Biotechnol. 2021 Jul 8;39(12):1563–73. doi: 10.1038/s41587-021-00968-7 (PMC8668435; doi:10.1038/s41587-021-00968-7)
Supplement: Supplementary file 1 — Supplementary Figs. 1–17 and Notes. [file 41587_2021_968_MOESM1_ESM.pdf]

nature portfolio

https://doi.org/10.1038/s41587-021-00968-7

Supplementary information

MaxDIA enables library-based and library-free data-independent acquisition proteomics

In the format provided by the authors and unedited

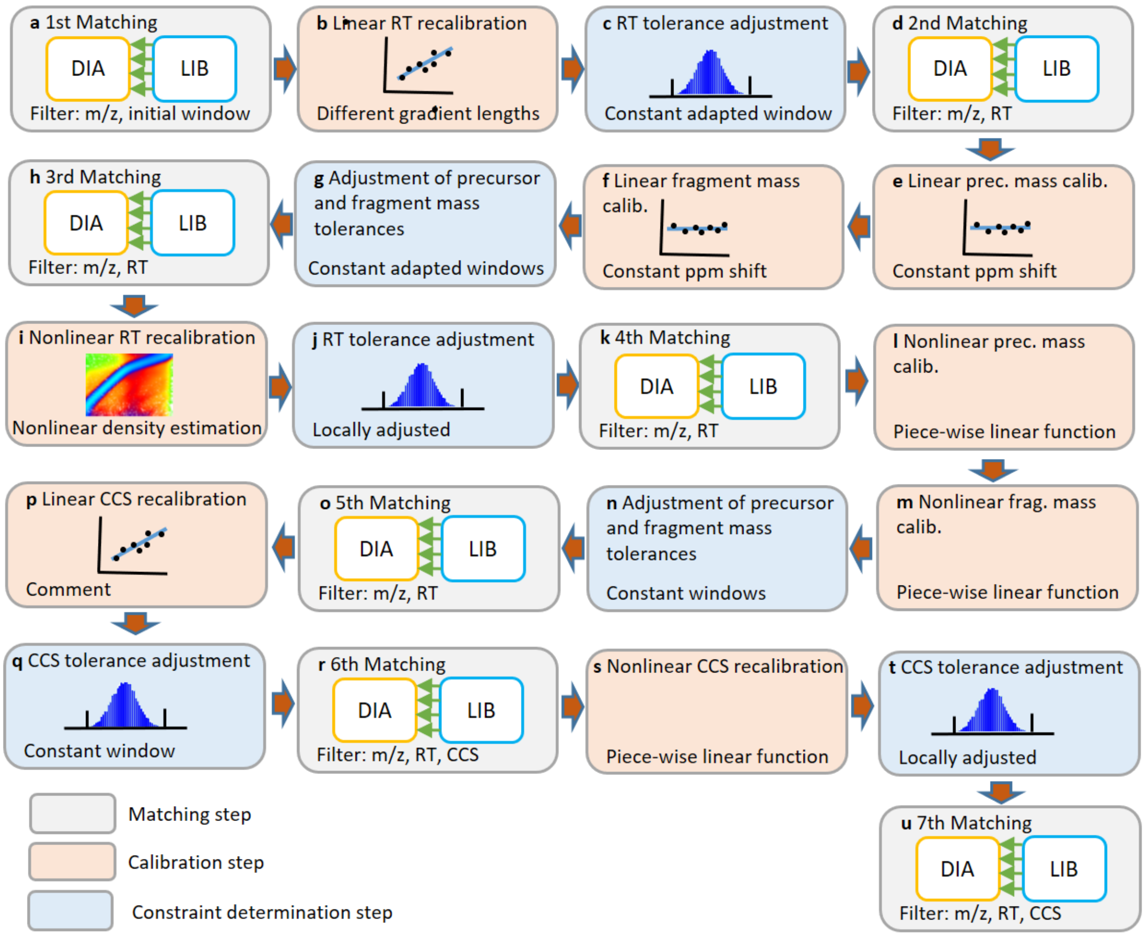

Supplementary Fig. 1: The bootstrap DIA workflow. This sequence of algorithmic steps is applied to each DIA sample vs. the whole library. A matching step is usually followed by a step in which a calibration function (e.g. precursor m/z recalibration function) is determined from the matches found in the previous step. Then constraints (e.g. m/z deviation windows) are updated for the next round of matching. The DDA samples constituting the library are assumed to be retention time (and ion mobility if applicable) aligned to each other. **a,** The first matching from the library spectra to the DIA sample is performed with initial m/z windows for precursor and fragments of 20 p.p.m. by default and without restrictions on retention times or collision cross sections. **b,** Based on these matches, a linear recalibration is calculated to adjust for different total gradient lengths of library and DIA samples. **c,** After the linear retention time calibration has been calculated and applied, a time window is calculated from the data, which defines the allowed retention time difference for the next step. **d,** The second matching still uses the initial m/z windows and in addition uses the time window determined in the previous step. **e,** Based on the matches of the previous step a linear precursor m/z shift in p.p.m. between the DIA sample and calculated peptide masses is determined. **f,** Similarly, a fragment m/z shift is calculated

from the data. **g,** Next, precursor and fragment m/z tolerances are calculated based on the distributions of m/z differences between DIA sample and theoretically calculated masses. **h,** The third matching uses adapted m/z and retention time windows which are applied to the linear calibrated data. **i,** The elimination of noise achieved by the adapted tolerances used in the matching in the previous step allows now to perform nonlinear retention tine calibration. **j,** A time dependent nonlinear allowed region is determined from the data. **k,** The fourth matching uses more stringent retention time constraints than the third matching, since it is applied to nonlinear calibrated data. **l,** Now a nonlinear calibration of precursor m/z values is determined from the data. This is done in a multivariate way, with a model for the mass error depending at least on m/z and retention time. For TOF data an intensity-dependent component is added and for timsTOF data another component depending on 1/K0. This is similar to the 'software lock mass' calibration in the DDA MaxQuant workflow. **m.** Similarly, fragment m/z are nonlinear recalibrated. **n,** New, more stringent precursor and fragment m/z tolerances are calculated from the distributions of mass errors. **o.** Another matching step with updated constraints is performed. **p,** A linear function for the recalibration of CCS values is calculated from the data, in case of ion mobility spectrometry. **q,** A tolerance window for the acceptance of CCS value deviations is calculated. **r,** A matching round with constraints on the CCS values is performed. **s,** A nonlinear CCS calibration function is determined. **t,** CCS tolerance is adapted to the nonlinear calibrated data. **u,** The final round of matching is performed without constraints on retention time and CCS values. Instead, these deviations are used as features in the XGBoost-based machine learning. Precursor and fragment masses are still filtered with hard windows for the deviations.

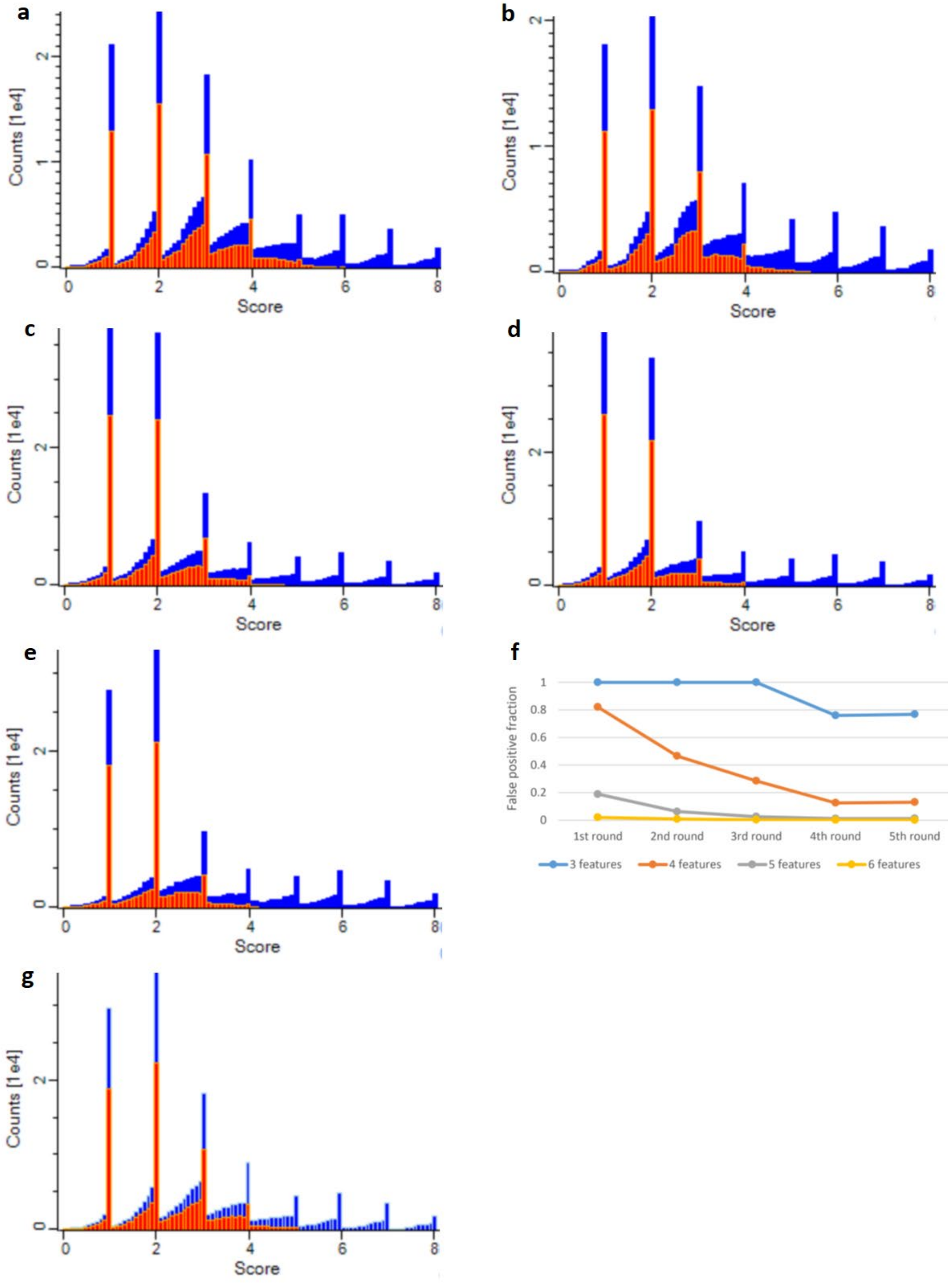

Supplementary Fig. 2: Score distributions along the bootstrap DIA workflow. Histograms of score distributions, separately for target and decoy hits after the different matching steps in the bootstrap DIA workflow. Target (blue) and decoy (red) distributions

are stacked on top of each other. A single run of the HepG2 Orbitrap dataset (DIA_13.raw) was used. **a,** Score histogram after the first matching step. (Step a in Supplementary Fig. 1.) No constraints on the retention time are used. Initial tolerances of 20 p,p,m. are applied to precursor and fragment mass matches. The spikes at integer score values correspond to matches in which all matching fragments hit exactly the apex of the peak in retention time direction. The peaks from one to four matching fragments are dominated by false positives, since these bins have half or even more decoy hits. Score values of six or above indicate correctness of the match since decoy hits are strongly suppressed. **b,** Score histogram after the second matching step. (Step d in Supplementary Fig. 1.) Retention time is filtered after linear retention time calibration between library and DIA sample and after determining a tolerance from the distribution of retention time differences. **c,** Score histogram after the third matching step. (Step h in Supplementary Fig. 1.) Linear ppm shifts are applied to precursor and fragment masses and mass tolerances are adapted accordingly. Scores larger than four indicate few false positives, **d,** Score histogram after the fourth matching step. (Step k in Supplementary Fig. 1.) **e,** Score histogram after the fifth matching step. (Step o in Supplementary Fig. 1.) in which nonlinear mass recalibrations have been applied to the data. **f,** Each profile shows the rate of false positive matches after each of the five different matching steps. The numbers are derived from the bins at integer values in the histograms of the previous panels. **g,** After all recalibrations have been applied, the final matching is done without constraints on retention times, but the mass constraints are kept. (The corresponding score distribution is displayed.) Instead the deviation from the calibrated retention time is offered as a feature to the machine learning for calculating an enhanced score. This strategy (hard mass cutoffs and soft, machine learning based, retention time cutoff) resulted in the highest number of identifications. Similarly, a soft cutoff is used for collision cross sections in ion mobility spectrometry data.

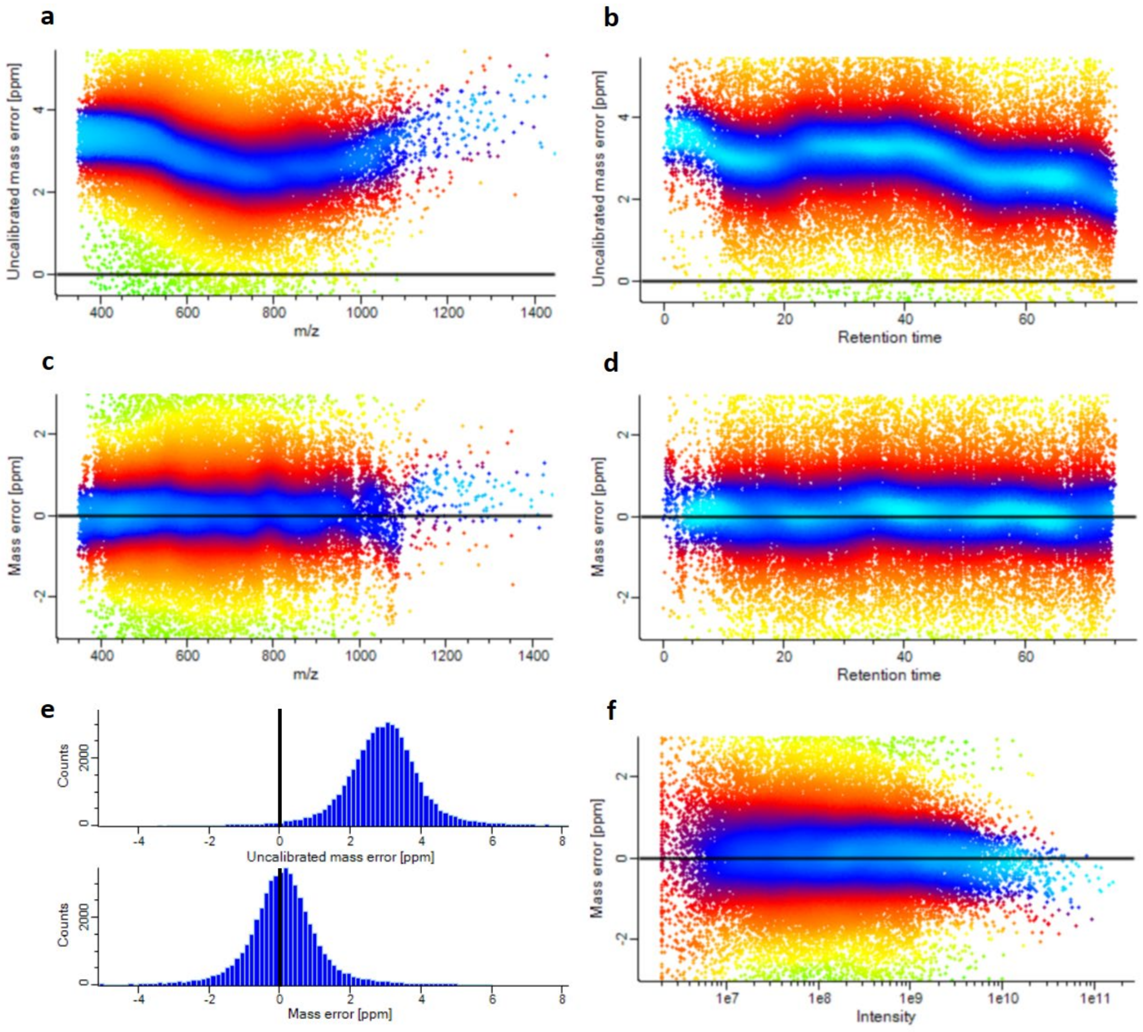

Supplementary Fig. 3: Nonlinear m/z recalibration of precursors. One consequence of the bootstrap DIA is that masses of precursors and fragments are nonlinearly recalibrated against theoretically calculated molecule masses. This replaces the software lock mass strategy used in DDA MaxQuant, which is based on a 'first search' with the Andromeda search engine to produce the recalibration curves. We use the same data as in Supplementary Fig. 2 to compare mass errors before and after recalibration. In all panels, data points are color coded according to the conditional data density. For this, the bivariate density of data points is divided by the marginal distribution on the x-axis. Blue signifies the region of highest conditional density. **a,** Mass error in p.p.m. of precursor ions as a function of m/z. **b,** Same precursor mass error as in panel a as a function of retention time. **c,d** Mass errors of panels a and b after recalibration through bootstrap DIA. The high-density regions are centered around 0 error. **e,** Histograms of precursor mass errors before and after recalibration. The medians of the error distributions are at 2.96 p.p.m. before and at 0.099 ppm after recalibration. The FWHM reduces from 1.92 to 1.61 p.p.m.. **f,** Dependency of the precursor mass error on logarithmic intensity. Interestingly, does the

distribution of mass error not depend much on the intensity, since the lines of constant density (constant color) run approximately horizontally.

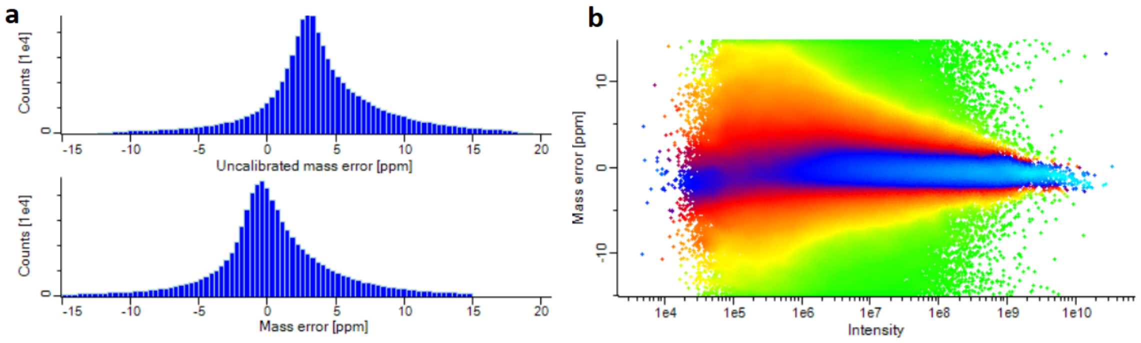

Supplementary Fig. 4: Nonlinear m/z recalibration of fragments. a, Histograms of fragment mass errors before and after recalibration. Since in this dataset, the statistical fluctuations are much larger for the fragment mass errors compared to the precursors, the correction of systematic errors is of less importance here. **b,** Dependency of the fragment mass error on logarithmic intensity. The distribution of mass errors gets wider towards lower intensities.

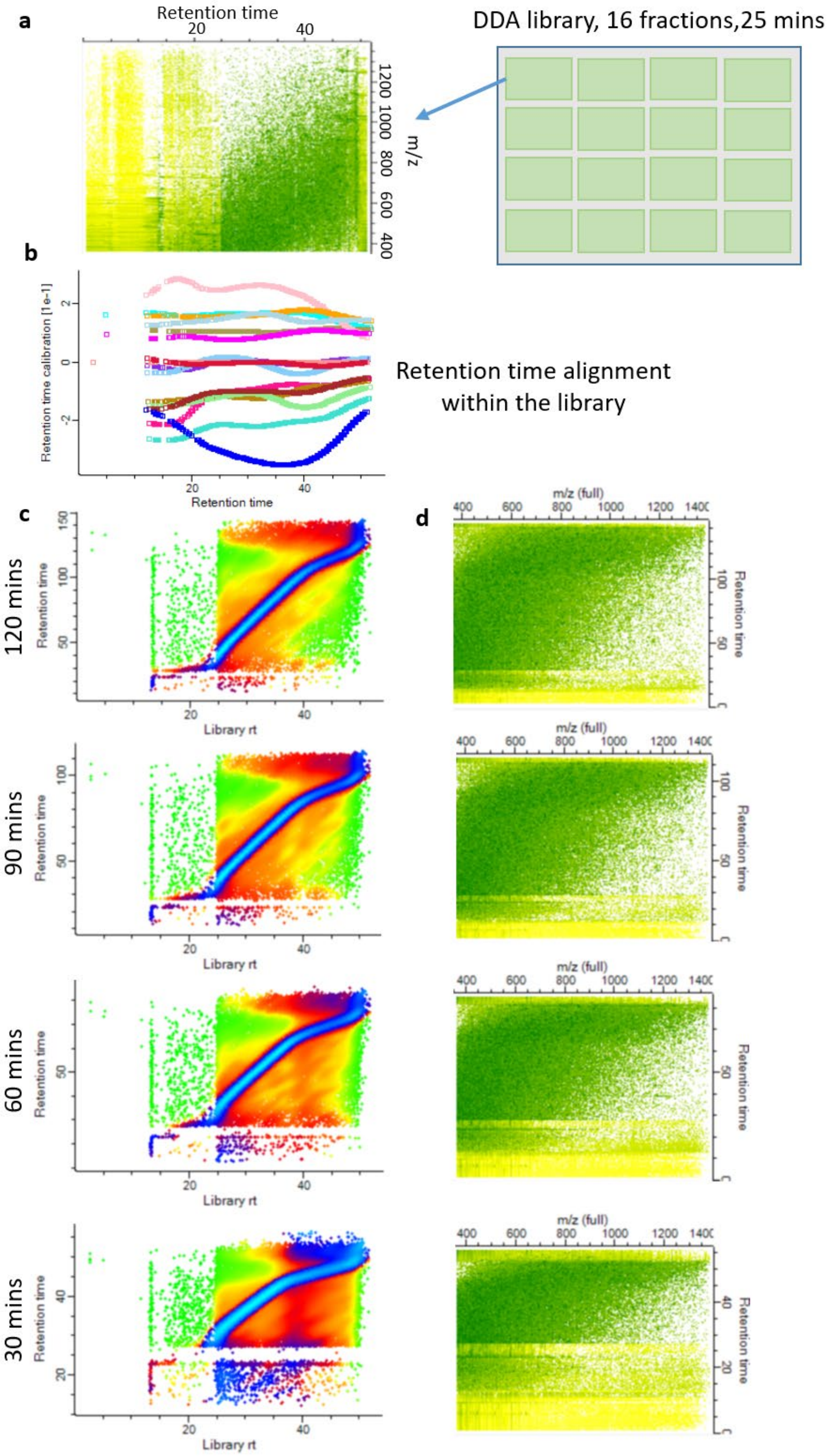

a
Retention time
20
40
DDA library, 16 fractions,25 mins
1200 1000 800 600 400
m/z
b
Retention time calibration [1e-1]
2
0
-2
Retention time alignment
within the library
20
40
Retention time
c
120 mins
Retention time
Library rt
90 mins
60 mins
30 mins
d
m/z (full)
400 600 800 1000 1200 1400
Retention time

Supplementary Fig. 5: Nonlinear retention time alignment between different gradients. a, A library of HeLa cell lysate was measured in 16 high-pH reversed phase peptide fractions with an active gradient time of 25 minutes. **b,** While analyzing the library in MaxQuant in DDA mode, retention times are aligned between the LC-MS runs in the library. **c,** Alignment of library retention times against for DIA samples with active gradient times of 120, 90, 60 and 30 minutes. **d,** Heat map views of the MS1 m/z-retention time planes of the respective DIA samples.

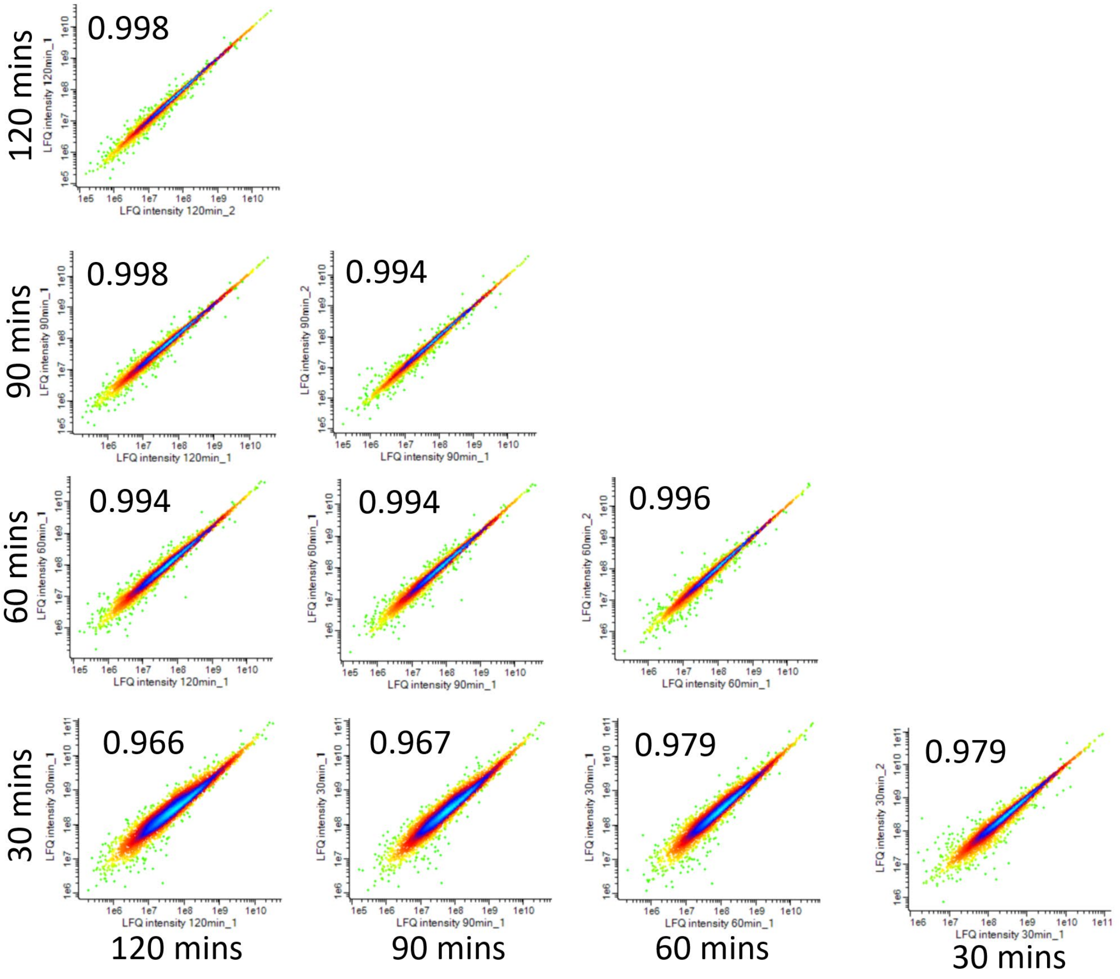

Supplementary Fig. 6: Nonlinear retention time alignment: LFQ after the alignment. Triangular matrix of scatter plots showing MaxLFQ quantification results between the four DIA samples with different gradients. The default value of 0.3 was used for the transfer q-value. The alignment enables precise quantification even between samples with vastly different gradients. On the diagonal, technical replicates with same gradients are shown. Pearson correlation coefficients between logarithmic LFQ intensities range from 0.998 for 120h gradients to 0.979 for 30h gradients. Throughout, quantification between non-equal gradients results in Pearson correlation values close to the one achieved with equal gradients of the respective shorter length.

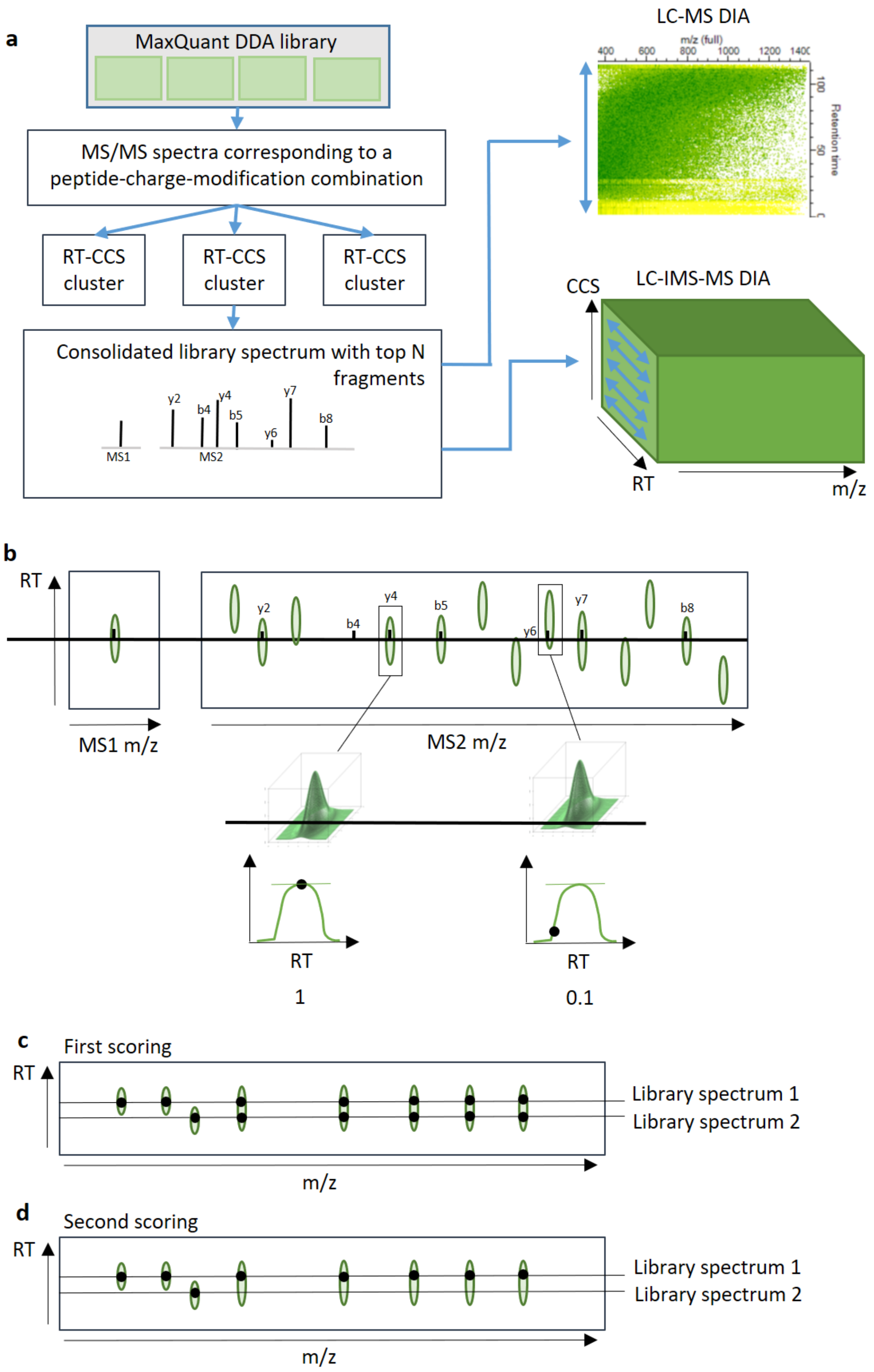
a
MaxQuant DDA library
MS/MS spectra corresponding to a peptide-charge-modification combination
RT-CCS cluster
RT-CCS cluster
RT-CCS cluster
Consolidated library spectrum with top N fragments
y2
y4
b4
b5
y7
y6
b8
MS1
MS2
LC-MS DIA
m/z (full)
400
600
800
1000
1200
Retention time
100
50
LC-IMS-MS DIA
CCS
RT
m/z
b
RT
y2
b4
y4
b5
y6
y7
b8
MS1 m/z
MS2 m/z
RT
RT
1
0.1
c
First scoring
RT
Library spectrum 1
Library spectrum 2
m/z
d
Second scoring
RT
Library spectrum 1
Library spectrum 2
m/z

Supplementary Fig. 7: Scoring library spectra against DIA samples. a, Libraries are collections of DDA samples analyzed with MaxQuant. MS/MS spectra from the library are first sub-divided into unique peptide-charge-modification combinations. Each such combination that has assigned more than one MS/MS spectrum to it is then clustered into retention time clusters. Prerequisite for this is that all library samples are retention-time aligned to each other. The idea is that if a peptide is eluting at more than one place in a gradient, it will be stored as multiple instances in the library with different retention times. This is feasible, since from the MaxQunat DDA analysis it is known how the peptides elute from their MS1 features. For data with ion mobility spectrometry this kind of library feature clustering is done in the two-dimensional space consisting of retention times and collision cross sections. A resulting cluster may still contain more than one MS/MS spectrum. In that case, the one with the highest Andromeda score is chosen. This spectrum is then filtered to the top-N most intense fragment peaks. These are then scored against the DIA sample. By default, is N = 7. We visit each retention time in a DIA LC-MS run and calculate the score which is defined below. The matching position is defined as the retention time at which the highest score is achieved. This highest value of the score is also defined as the matching score of this library spectrum to the DIA sample. For ion mobility spectrometry, this score maximization takes place in the two-dimensional space of all retention time and ion mobility value pairs. **b,** For calculating the score of a library spectrum at a certain retention time (and CCS value) in the DIA sample, one first searches with a given mass tolerance for 3D/4D features that match the precursor and the N (typically = 7) top fragment peaks. For each spectrum mass that matches a feature in the DIA sample we calculate the apex fraction which is the ratio of the intensity at the current retention time to the maximum peak intensity. To obtain the score, we sum up the apex fractions for the precursor (in case one was matched) and the matching fragments. **c,** So far the scoring was done independently for each consolidated library spectrum. This can lead to multiple usages of a DIA feature in several library matches. **d,** To prohibit over-interpretation, we perform a second round of scoring. This time we put the library spectra in descending order according to the score they achieved in the first round of scoring. The same procedure is repeated, but now it is remembered which features in the DIA sample (precursors and fragments) have already been assigned and these will be prohibited from being assigned a second time. Note that an MS1 precursor match is not required but contributes the same way to the total score as each fragment does.

a

No.	Feature
1	Score
2	Pearson intensity correlation
3	Spearman intensity correlation
4	DIA range index
5	Peptide sequence length
6	Precursor charge
7	Retention time
8	Retention time error
9	1/K0
10	1/K0 error
11	Number of (unfiltered) library fragments
12	Andromeda score of library spectrum
13	Number of missed cleavages
14	Number of modifications
15	Sample fragment overlap
16	Number of fragments found
17	Library index (in case of multiple libraries)
18	Precursor mass (calculated)
19	Precursor match score
20	Precursor mass error [ppm]
21	Precursor RT peak length (number of scans)
22	Precursor found (yes/no)
23	Precursor has isotope pattern (yes/no)
24	Mass difference to range min
25	Mass difference to range max

b

No.	Feature
1	Fragment match score
2	Fragment mass error [ppm]
3	Fragment RT peak length (number of scans)
4	Fragment found (yes/no)
5	Fragment has isotope pattern (yes/no)

c

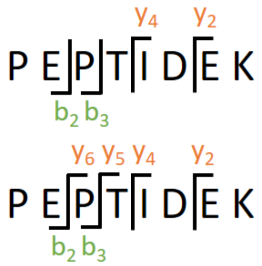

d

Rank	Feature	Gain
1	Precursor has isotope pattern	73.1
2	Score	42.7
3	Retention time error	13.4
4	Number of modifications	12.9
5	Number of missed cleavages	11.4
6	Fragment has isotope pattern (1)	10.0
7	Precursor mass error [ppm]	9.0
8	Precursor charge	7.3
9	Sample fragment overlap	7.2
10	Fragment has isotope pattern (2)	6.8

e

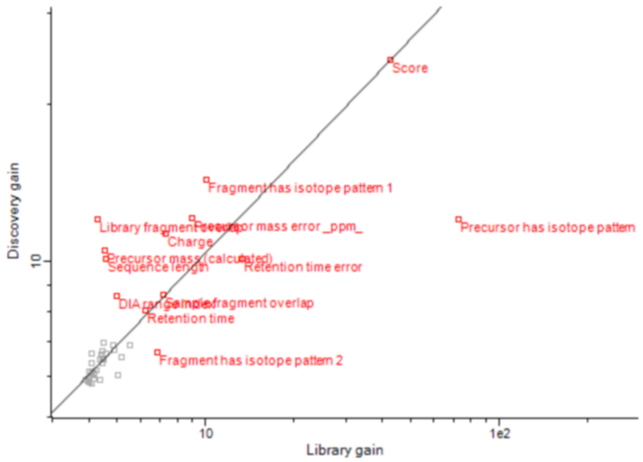

Supplementary Fig. 8: Feature space for the machine learning-based score. a, 25 'single' features for the feature matrix for calculating the machine learning score. Features 2 and 3 are correlations between the fragment intensities found in the DIA sample and the library fragment intensities. Feature 9 specifies the collision cross section value, in case ion mobility data is available. Feature 11 is the number of fragments in the library spectrum

before filtering for the top intense peaks. Feature 15 is explained in panel c. Feature 19 quantifies how close to its apex the precursor was hit. Feature 22 defines if the precursor was found in the MS1 data and feature 23 specifies whether an isotope pattern was seen. Features 24 and 25 quantify how close the peptide m/z is to the edges of the isolation window. **b,** Machine learning features derived from fragments. Feature 1 quantifies how close to its apex the fragment was hit. Feature 4 defines if the fragment was found and feature 5 specifies whether an isotope pattern was seen for it. By default, 7 top intense fragments are considered for identification which results in a 25 + 7 * 5 = 60 dimensional feature space in total. **c,** Explanation of the fragment overlap feature. The first peptide has a fragment overlap of 0 since the y and b ion series are not overlapping. The second peptide has overlapping y and b series and hence is its fragment overlap greater than 0. **d,** List of the top 10 features ranked by importance according to XGBoost 'gain'. Even more important than the score is whether the precursor had an isotope pattern or is a single feature. Interestingly, the absence or presence of the MS1 precursor did not make it into the top ten most relevant features. **e,** Log-log scatter plot of feature importance according to XGBoost 'gain' for library against discovery mode. To guide the eye, we drew a straight line from the cloud of non-important features in the lower left corner to the raw score, which is expected to be of high relevance for the classification. Whether the precursor feature has an isotope pattern became much less important in the discovery mode. Features that are correlated with peptide length and charge became more important in discovery mode, presumably since the length and charge distributions of predicted spectra in the in silico library are significantly different from these distributions for peptides that are detectable in the DIA samples.

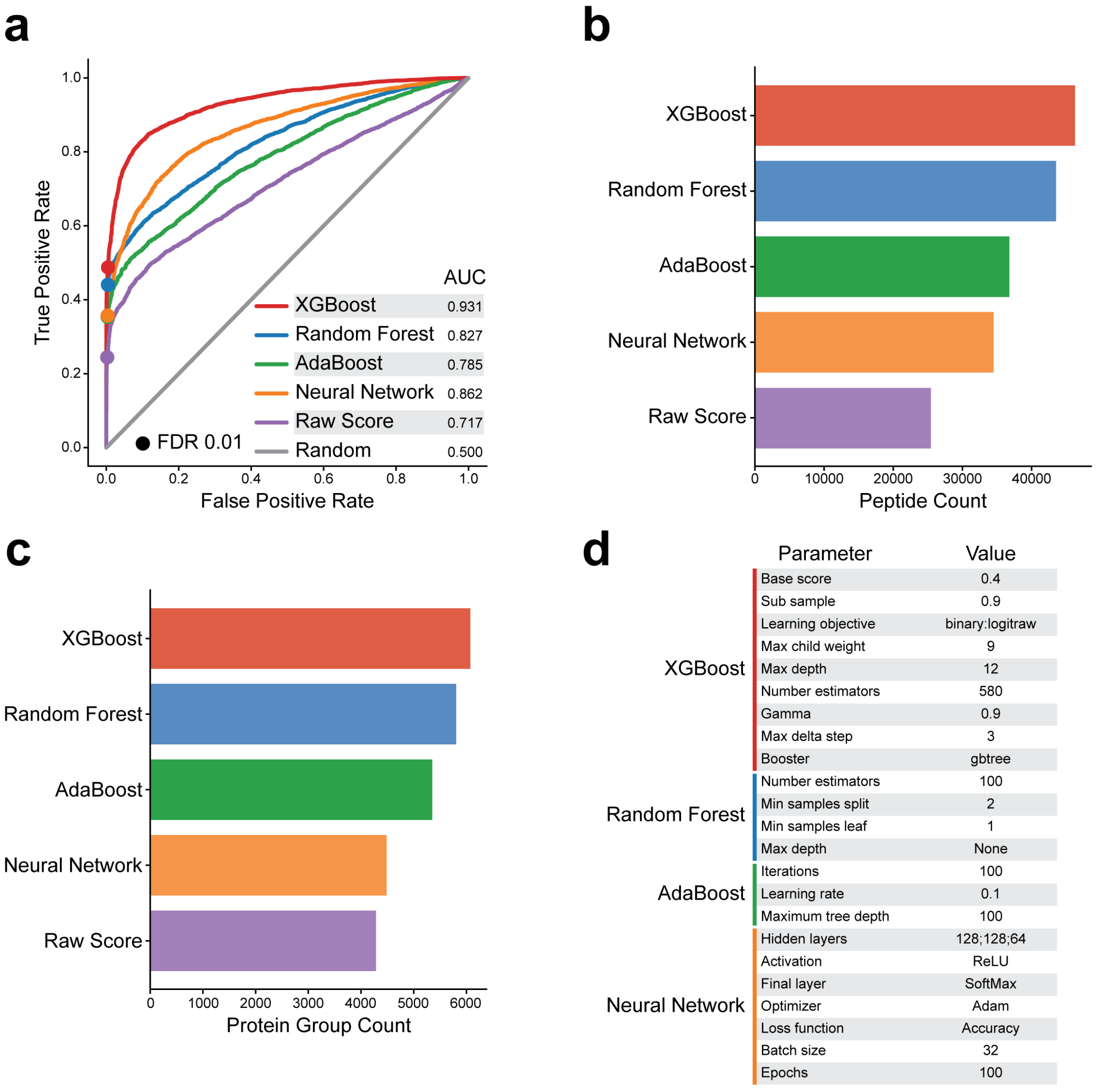

Supplementary Fig. 9: Comparison between different classification methods. We compared XGBoost, random forests, AdaBoost and fully connected multi-hidden layer neural networks to using the raw score. We tuned meta-parameters to its optimal value if applicable. **a,** ROC curves for the five classification methods. XGBoost has the highest area under the curve. **b,** Number of identified peptides when using each of the four classification Methods or the raw score in MaxDIA. XGBoost results in the highest number of peptide identifications. **c,** Number of identified protein groups when using each of the four classification Methods or the raw score in MaxDIA. XGBoost results in the highest number of peptide identifications. **d,** Optimal values of classification algorithm parameters found in grid searches.

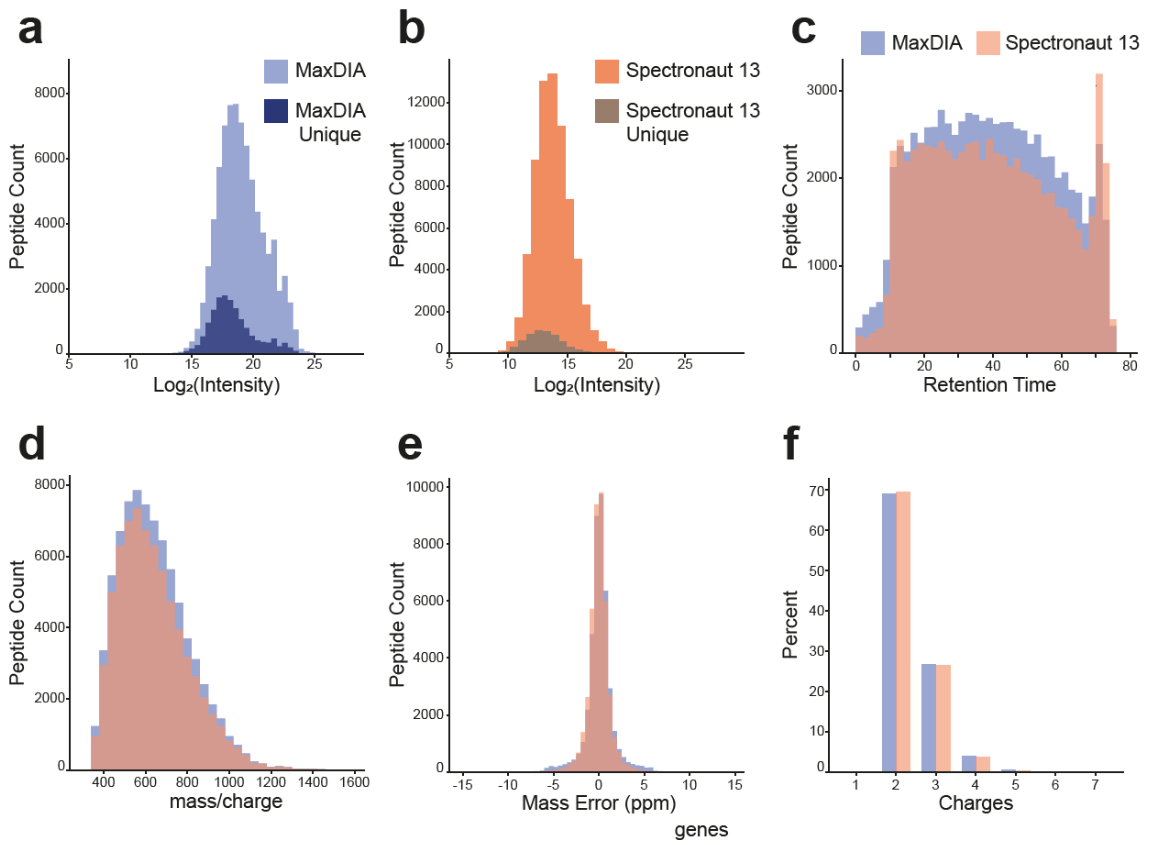

g

		MaxDIA	Spectronaut 13	Spectronaut 14: default	Spectronaut 14: inverse data base	Spectronaut 14: profiling strategy on	Spectronaut 14: 10 precursors 10 peptides
genes		6238	6015	6304	6242	6304	6297
peptides		67893	63223	64200	64232	64200	64146
coefficient of variation	Q1	0.049	0.077	0.080	0.079	0.080	0.81
	Q2	0.071	0.108	0.112	0.111	0.113	0.133
	Q3	0.105	0.178	0.195	0.188	0.197	0.229

Supplementary Fig. 10: Comparison of peptide properties. We compare different properties of identified peptides in the benchmark datasets between MaxDIA and Spectronaut. **a,** Logarithmic distribution of the MaxQuant intensities of all peptides found by MaxDIA is shown (light blue). Peptides uniquely found by MaxDIA are highlighted in dark blue. These are biased towards lower intensites. **b,** Logarithmic distribution of the Spectronaut intensities of all peptides found by Spectronaut (orange) with the ones found uniquely by Spectronaut highlighted in brown. Unique peptides are biased towards lower intensities here as well, but they are less in total compared to panel a. Note that the intensity ranges in panels a and b differ, since these are computed differently in the two programs. For instance, peptide intensities in MaxDIA are calculated from MS1 features. (Please note that this is not the case for the protein-level MaxLFQ intensities, which are by default

hybrid MS1-MS2.) **c,** Distributions of retention times of peptides identified by MaxDIA and Spectronaut. **d,** Distributions of precursor mass-to-charge ratios of peptides identified by MaxDIA and Spectronaut. **e,** Distributions of precursor mass errors in p.p.m. of peptides identified by MaxDIA and Spectronaut. **f,** Distributions of charges of peptides identified by MaxDIA and Spectronaut. **g,** Detailed comparison of identification results between MaxDIA, Spectronaut 13 and Spectronaut 14. For the latter we tested the impact of changing a set of parameters one by one from their default values on the result. In particular, we used the inverse database, we set profiling strategy to 'on' and we used 10 precursors and 10 peptides. None of these settings had a major impact on the results or changed the overall conclusions.

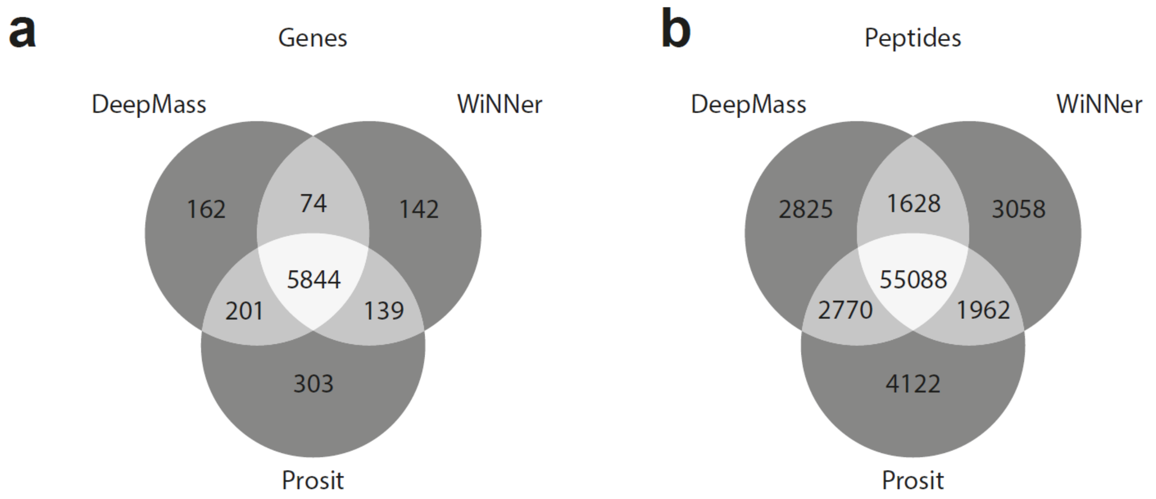

Supplementary Fig. 11: Comparison of identification results in discovery mode obtained with DeepMass:Prism, wiNNer and PROSIT. In order to study the sensitivity of identification results in discovery mode towards the machine learning algorithm used for predicting the MS/MS spectra, we repeated all calculations using the predictions of two other state of the art prediction models, winner and PROSIT, both used with default settings. In PROSIT the optimal collision energy was determined and found to be 32. Instructions for preparing in-silico libraries with DeepMass:Prism, winner and PROSIT can be found at https://github.com/cox-labs/DIAtools/blob/main/Misc/MLprediction/README.md#MLprediction. **a,** Comparison of results on gene level. For better comparability, we mapped the identified protein groups of the three approaches to Entrez gene identifiers. The vast majority of genes (protein groups) has been identified in all three approaches with a very slight lead in the collision energy-aware PROSIT identifications. **b,** Same as a but with comparison on the peptide level.

a

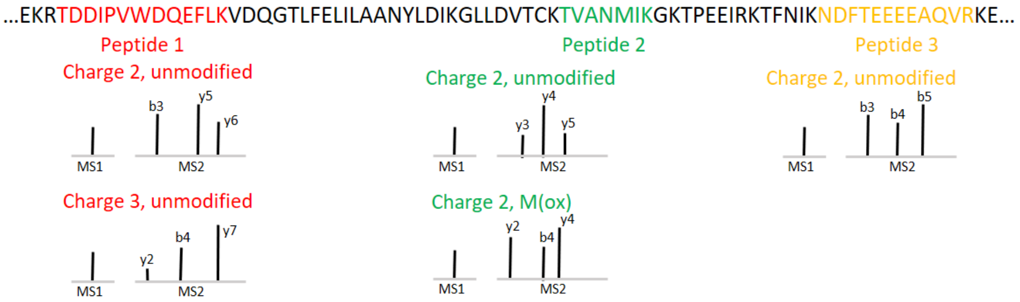

b

Combi nation	Sequence	Charge	Modification	Ion	Sample 1	Sample 2	Sample 3	Sample 4
1	Peptide 1	2	Unmodified	Precursor	*Int 1,1*	*Int 1,2*	*Int 1,3*	*Int 1,4*
2	Peptide 1	2	Unmodified	1st fragment	*Int 2,1*	*Int 2,2*	*Int 2,3*	*Int 2,4*
3	Peptide 1	2	Unmodified	2nd fragment	*Int 3,1*	*Int 3,2*	*Int 3,3*	*Int 3,4*
4	Peptide 1	2	Unmodified	3rd fragment	*Int 4,1*	*Int 4,2*	*Int 4,3*	*Int 4,4*
5	Peptide 1	3	Unmodified	Precursor	*Int 5,1*	*Int 5,2*	*Int 5,3*	*Int 5,4*
6	Peptide 1	3	Unmodified	1st fragment	*Int 6,1*	*Int 6,2*	*Int 6,3*	*Int 6,4*
7	Peptide 1	3	Unmodified	2nd fragment	*Int 7,1*	*Int 7,2*	*Int 7,3*	*Int 7,4*
8	Peptide 1	3	Unmodified	3rd fragment	*Int 8,1*	*Int 8,2*	*Int 8,3*	*Int 8,4*
9	Peptide 2	2	Unmodified	Precursor	*Int 9,1*	*Int 9,2*	*Int 9,3*	*Int 9,4*
10	Peptide 2	2	Unmodified	1st fragment	*Int 10,1*	*Int 10,2*	*Int 10,3*	*Int 10,4*
11	Peptide 2	2	Unmodified	2nd fragment	*Int 11,1*	*Int 11,2*	*Int 11,3*	*Int 11,4*
12	Peptide 2	2	Unmodified	3rd fragment	*Int 12,1*	*Int 12,2*	*Int 12,3*	*Int 12,4*
13	Peptide 2	2	M(ox)	Precursor	*Int 13,1*	*Int 13,2*	*Int 13,3*	*Int 13,4*
14	Peptide 2	2	M(ox)	1st fragment	*Int 14,1*	*Int 14,2*	*Int 14,3*	*Int 14,4*
15	Peptide 2	2	M(ox)	2nd fragment	*Int 15,1*	*Int 15,2*	*Int 15,3*	*Int 15,4*
16	Peptide 2	2	M(ox)	3rd fragment	*Int 16,1*	*Int 16,2*	*Int 16,3*	*Int 16,4*
17	Peptide 3	2	Unmodified	Precursor	*Int 17,1*	*Int 17,2*	*Int 17,3*	*Int 17,4*
18	Peptide 3	2	Unmodified	1st fragment	*Int 18,1*	*Int 18,2*	*Int 18,3*	*Int 18,4*
19	Peptide 3	2	Unmodified	2nd fragment	*Int 19,1*	*Int 19,2*	*Int 19,3*	*Int 19,4*
20	Peptide 3	2	Unmodified	3rd fragment	*Int 20,1*	*Int 20,2*	*Int 20,3*	*Int 20,4*

c

Sample 2	Ratio 2,1		
Sample 3	Ratio 3,1	Ratio 3,2	
Sample 4	Ratio 4,1	Ratio 4,2	Ratio 4,3
	Sample 1	Sample 2	Sample 3

d

Ratio 2,1 = LFQ intensity 2 / LFQ intensity 1

Ratio 3,1 = LFQ intensity 3 / LFQ intensity 1

Ratio 4,1 = LFQ intensity 4 / LFQ intensity 1

Ratio 3,2 = LFQ intensity 3 / LFQ intensity 2

Ratio 4,2 = LFQ intensity 4 / LFQ intensity 2

Ratio 4,3 = LFQ intensity 4 / LFQ intensity 3

e

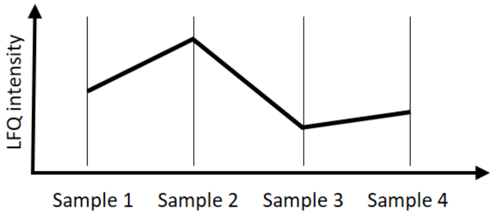

Supplementary Fig. 12: MaxLFQ algorithm for DIA. The conventional MaxLFQ algorithm for DDA consists of two parts, feature intensity normalization and protein quantification. While in the adaptation to DIA the normalization part did not change, the quantification was adapted to accommodate signals contributing from precursor and fragment features. **a,** As an example we use the protein sequence of UniProt entry P07327. Three peptides were identified, Peptide 1, unmodified with charge 2 and 3, Peptide 2, unmodified and with an oxidation of methionine, and Peptide 3, only unmodified with charge 2. These five peptide, charge and modification combinations are treated as independent intensities in the protein quantification, as was already the case in the DDA version of MaxLFQ. In DIA, also the different types of ions, precursors and fragments, are treated as separate signals. Feeding these as independent 'channels' into MaxLFQ is a natural way of implementing hybrid precursor-fragment quantification. For every combination of peptide, charge and modifications, we take the top N intense fragment peaks over the whole dataset. These N annotations are then used in every spectrum of this type for quantification. In the example we chose N = 3 for simplicity, although N is a user-definable parameter and much larger by default. (See Supplementary Fig. 13a for the influence of N on the quantification accuracy.) **b,** In the example from panel a with five peptide-charge-modification combinations and N = 3 we end up with 20 peptide-charge-modification-ion combinations. We assume that data for four samples was acquired. Then we have for this protein 20 intensity profiles over the four samples. Those intensities in this matrix which are zero we call missing, since they cannot be used for calculating ratios between samples. **c,** Next we calculate protein ratios between all pairs of samples to fill the lower triangular matrix indicated in the figure. 'Ratio 2,1' is the median of all ratios calculated from the intensities in the columns 'Sample 1' and 'Sample 2' in panel b. These are 20 if all values are present but can be less due to missing values. If the number of peptide-charge-modification combinations for which ratios can be calculated is less than the parameter 'LFQ min. ratio count' the corresponding ratio in the triangular matrix will be missing. **d,** For each ratio in panel c that is not missing we obtain one equation for the determination of the four LFQ intensities. (One for each sample.) This system of equations is usually over-determined and a least-squares best fit is obtained. **e.** Result of this operation is the profile of non-negative LFQ intensities over the four samples.

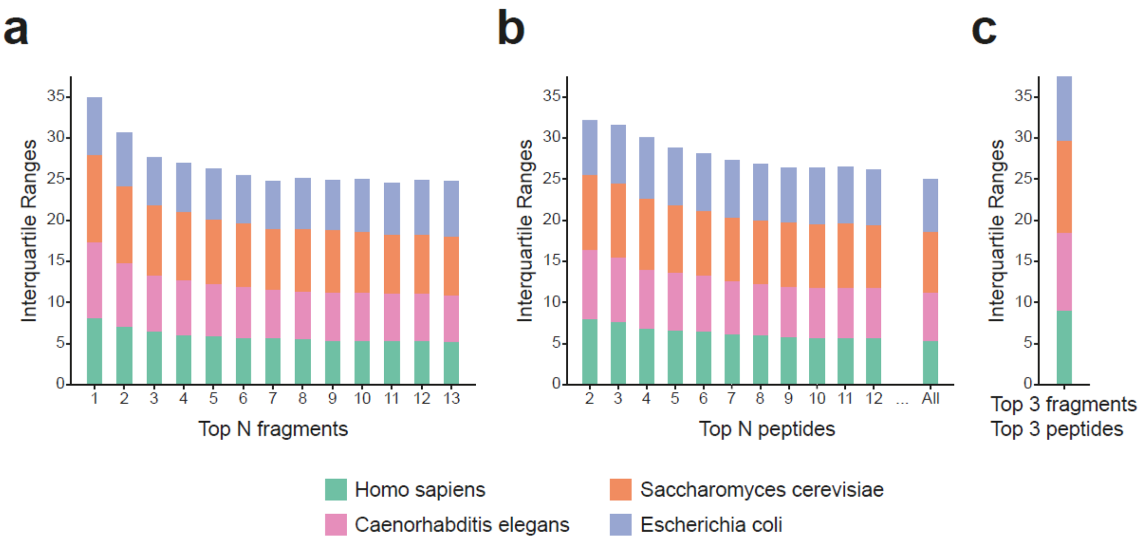

Supplementary Fig. 13: Optimization of number of top fragments and peptides. a, Summed inter-quartile ranges for the four-species benchmark dataset by Bruderer et al. as a function of the number of top intense fragments used for quantification. The accuracy is increasing with rising number of fragments and plateauing around seven fragments after which no noticeable improvement happens. The default value of 0.3 was used for the transfer q-value. **b,** Same as in panel a but optimizing the number of top intense peptides used for quantification. The more peptides are taken, the higher is the quantification accuracy. **c,** Same as in panels a and b but filtering for top 3 intense peptides and top 3 intense fragments simultaneously.

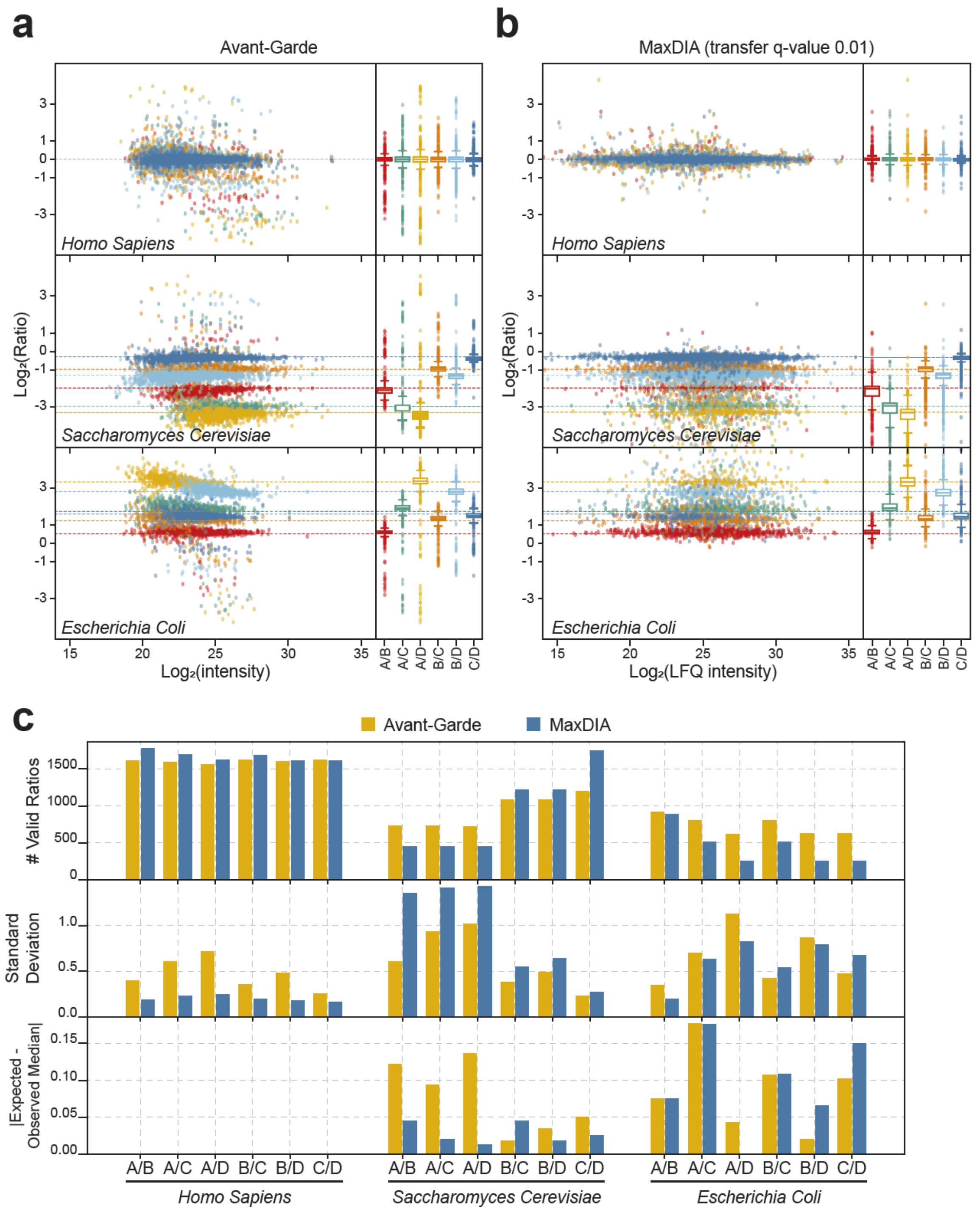

Supplementary Fig. 14: Comparison to Avant-garde filtered quantification. In order to judge how the accuracy of protein quantification with MaxLFQ for DIA compares to methods that explicitly filter the data for interfered transitions we use a dataset from Vaca Jacome et al. (Nature Methods, 2020) called 'Extended benchmarking DIA dataset' in the publication. There it was analyzed with the Skyline software and curated by Avant-garde. We analyzed the same data with MaxDIA and for comparison mapped MaxLFQ intensities

to Entrez gene identifiers. For the Avant-garde results taken from the publication, the median was taken over all peptide-level logarithmic ratios that were mapped to a gene identifier. All ratios were globally normalized such that the median of all the human log ratios is at zero. All box plots indicate the median and the first and third quartile as box ends. Whiskers are positioned 1.5 box lengths away from the box ends. **a,** Gene level ratios derived from the peptide-level ratios provided in the Avant-garde publication as a function of log(Intensity). 18 sub-populations of proteins (genes) exist with a defined expected ratio. Several outlier ratios are present at large deviations and some of the sub-populations show systematic trends with Log(Intensity). **b,** Same as in panel a but for MaxLFQ ratios. **c,** Comparisons of performance measures between Avant-garde and MaxDIA results. For all 18 sub-populations. The population-wise standard deviations are about half as low in MaxQuant results for the *H. sapiens* ratios. For the *S. cerevisiae* ratios tend to have a lower standard deviation with Avant-garde while there is no clear trend in the standard deviations of the *E. coli* ratios.

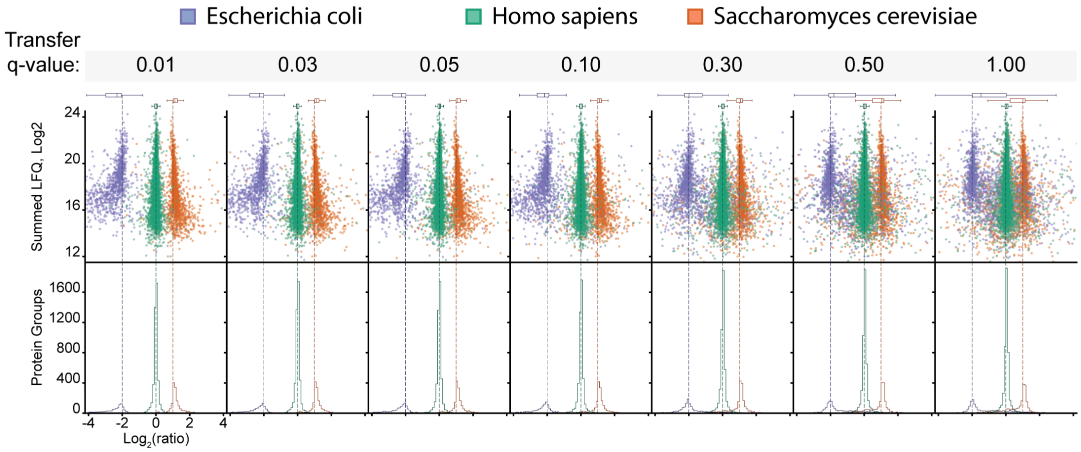

Transfer q-value	0.01	0.03	0.05	0.10	0.30	0.50	1.00
Valid LFQ ratios	1061 4523 1598	1130 4574 1672	1178 4595 1712	1284 4644 1787	1643 4775 2025	1679 4821 2176	1752 4831 2289
Valid Intensity ratios	1393 5436 2250	1448 5443 2306	1493 5445 2328	1580 5470 2377	1754 5520 2495	1837 5535 2575	1878 5537 2624
Summed IQR	1.30	1.20	1.16	1.08	1.56	2.41	3.04
Summed Absolute Error	0.43	0.38	0.34	0.25	0.11	0.29	0.58

Supplementary Fig. 15: Scanning through values for the transfer q.value. We analyzed the Bruker timsTOF pro three-species benchmark data using a range of values for the transfer q-value between 0.01 and 1. We provide summed inter-quartile ranges of species-specific ratio distributions as a measure of variability. Summed absolute errors are the deviations of the expected value for each species. The box plots are based on the numbers of data points given in the tables below the respective plot (Valid LFQ ratios). All box plots indicate the median and the first and third quartile as box ends. Whiskers are positioned 1.5 box lengths away from the box ends.

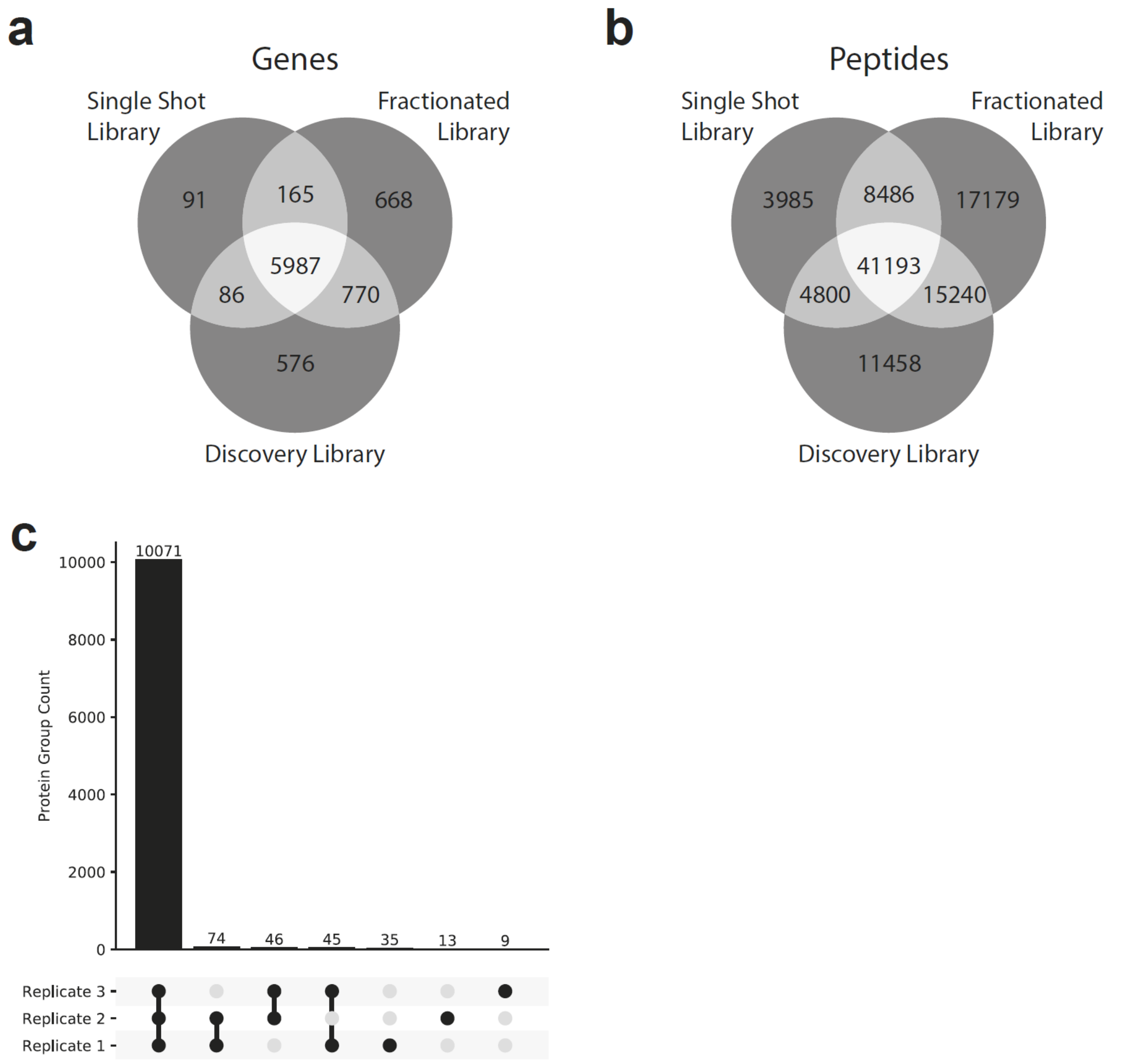

Supplementary Fig. 16: Single-shot BoxCar samples. a, Venn diagram of protein identifications mapped to Entrez gene identifiers for the single shot BoxCar DIA samples using three different library approaches. In particular, comparing protein identifications between fractionated library and discovery approach shows good agreement of results. **b,** Same as in panel a but comparing peptide-level identifications. **c,** Venn diagram-like comparison of replicate-specific identifications in the fractionated BoxCar DIA samples analyzed in discovery mode. Only very few protein groups were not identified in all three replicates.

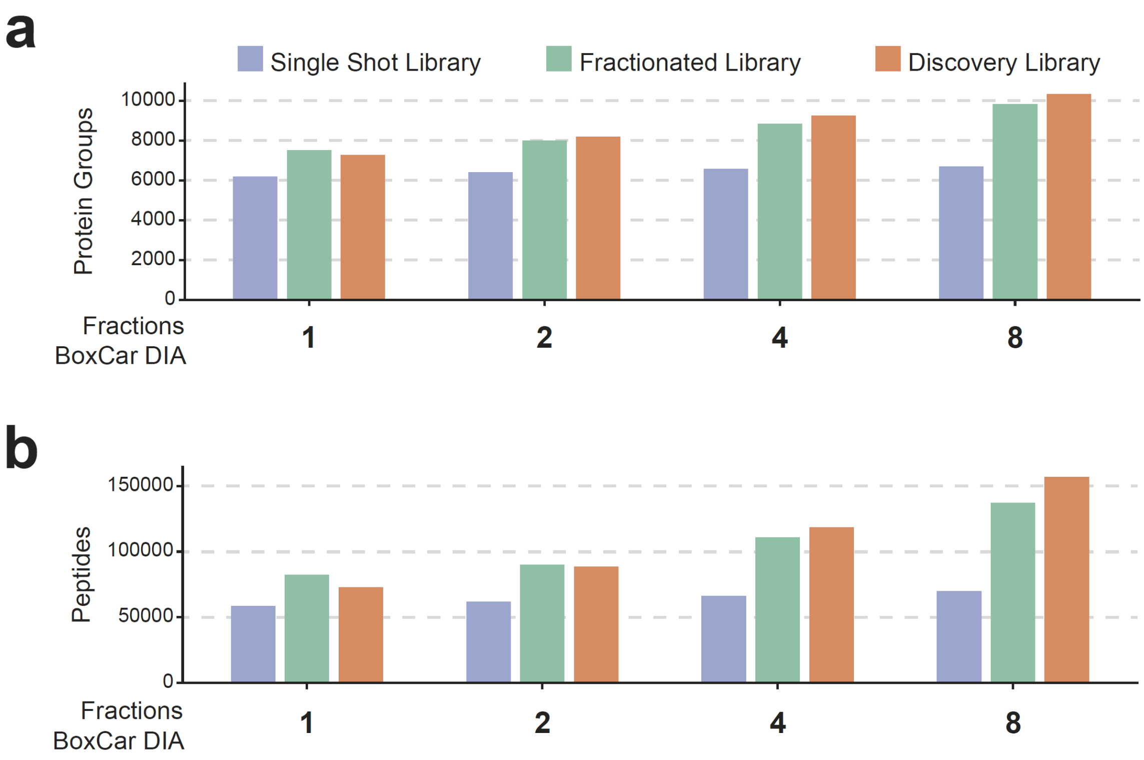

Supplementary Fig. 17: Dependence of identifications on the number of fractions. a, DIA samples were fractionated into one, two, four and eight fractions and analyzed with single-shot, fractionated and discovery library, similarly as in Figure 6. The number of identified protein groups is indicated for each of these cases. While the number of protein groups is not increasing much with the fractions when using a single shot library, there is a linear increase with the discovery library. **b,** Same as a but showing the number of identified peptides.

Supplementary notes to 'MaxDIA enables library-based and library-free data-independent acquisition proteomics' by Sinitcyn et al.

How to run MaxDIA in library mode

Summary: In order to enable MaxDIA for your DIA runs, after loading your mass spectrometry output data (raw data) into MaxQuant and setting your experiment design and the number of threads you'd like to utilize for your MaxQuant run, you can select either "Max DIA", "TIMS MaxDIA" or "BoxCar MaxDIA" from the "Type" menu within the "Group-specific parameters". Doing so will bring up a menu where you can specify your library files. These files include the peptide, evidence and msms text files from your DDA MaxQuant runs.

Note: To be able to run MaxQuant, .NET Core 2.1 needs to be installed. Please visit https://dotnet.microsoft.com/download/dotnet-core/2.1 and install the SDK x64."

Steps:

1. Using your internet browser, navigate to https://maxquant.org/

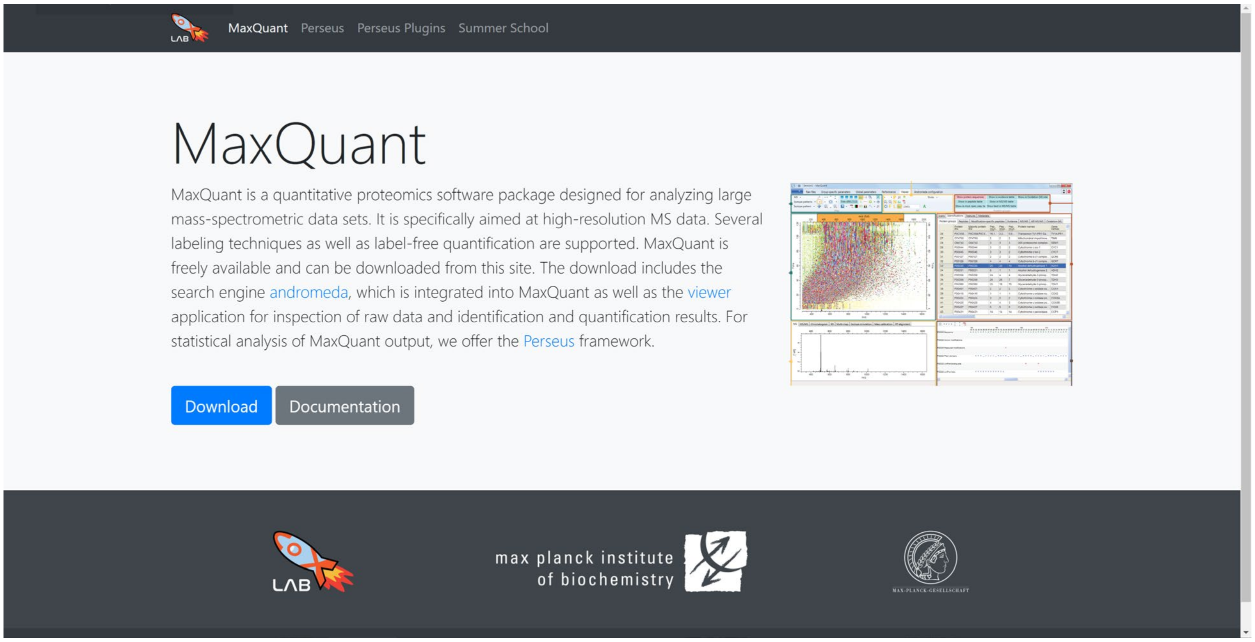

2. Click on the blue "Download" button to navigate to the download form.

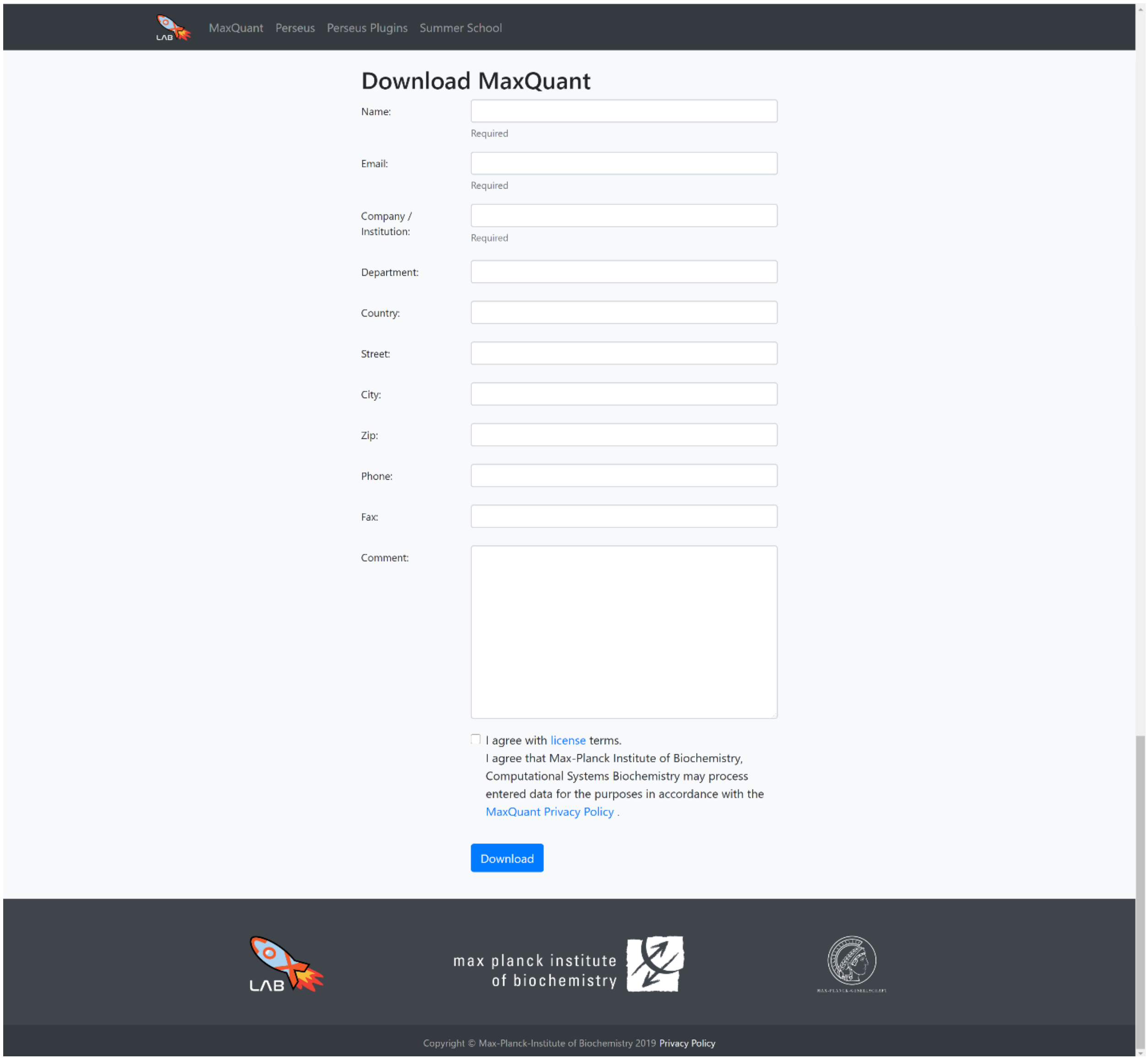

3. Fill in the form with your details and click on the check box at the end of the form to confirm your agreement with the MaxQuant license terms.
4. Click on the blue "Download" button to download MaxQuant.
5. Navigate to your downloads folder on your PC, where the zipped MaxQuant folder has been downloaded to.

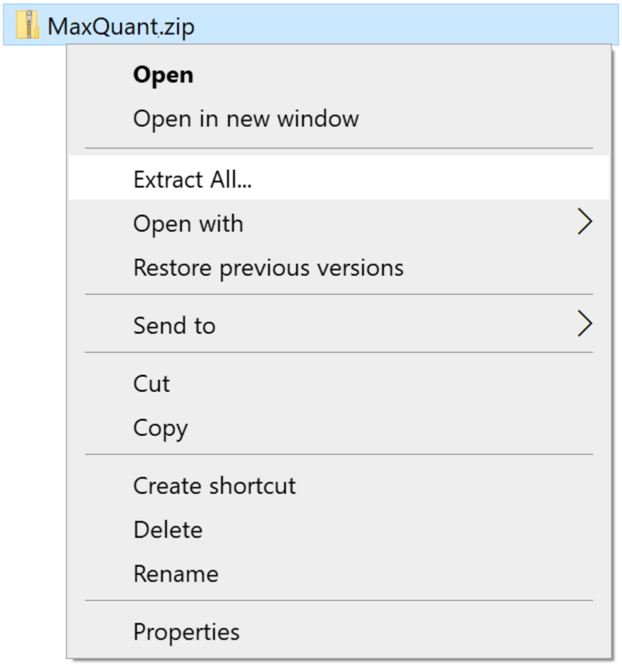

6. Extract the contents of the zipped MaxQuant folder you downloaded.

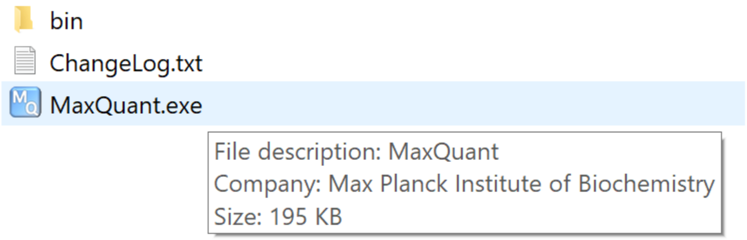

7. After extraction, open the extracted MaxQuant folder and double click on MaxQuant.exe to run MaxQuant.

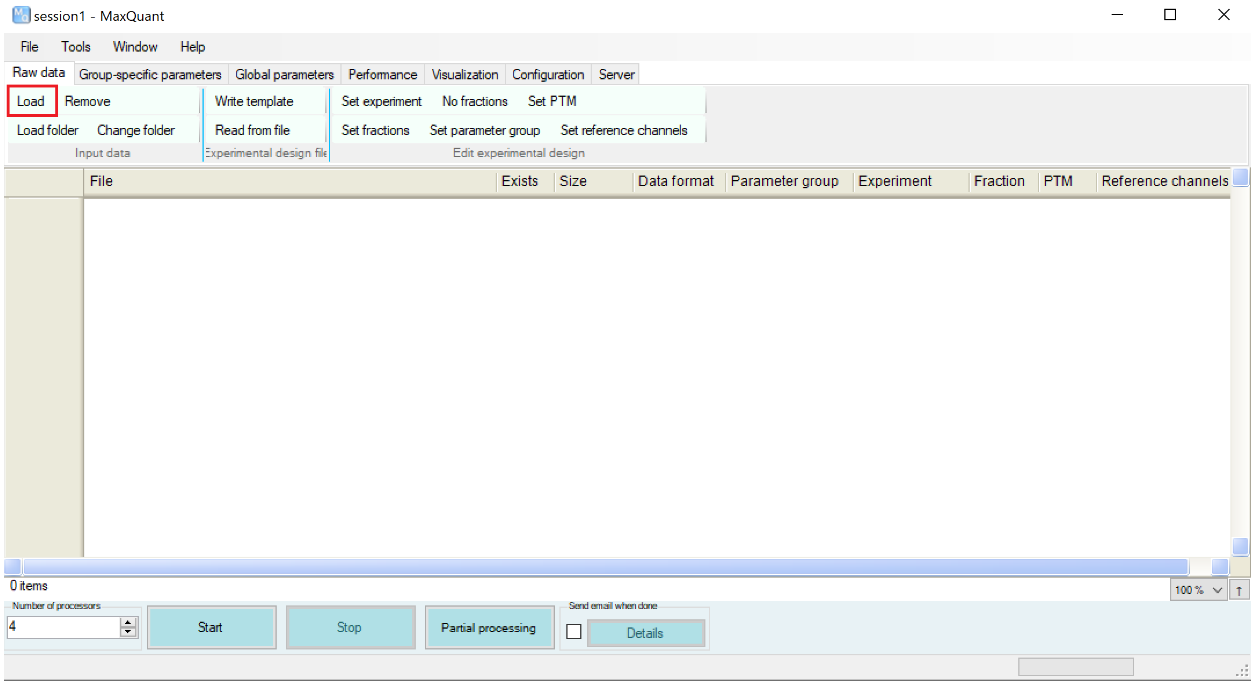

8. Click on the “Load” button to load your mass spectrometry output data (raw data) into MaxQuant.

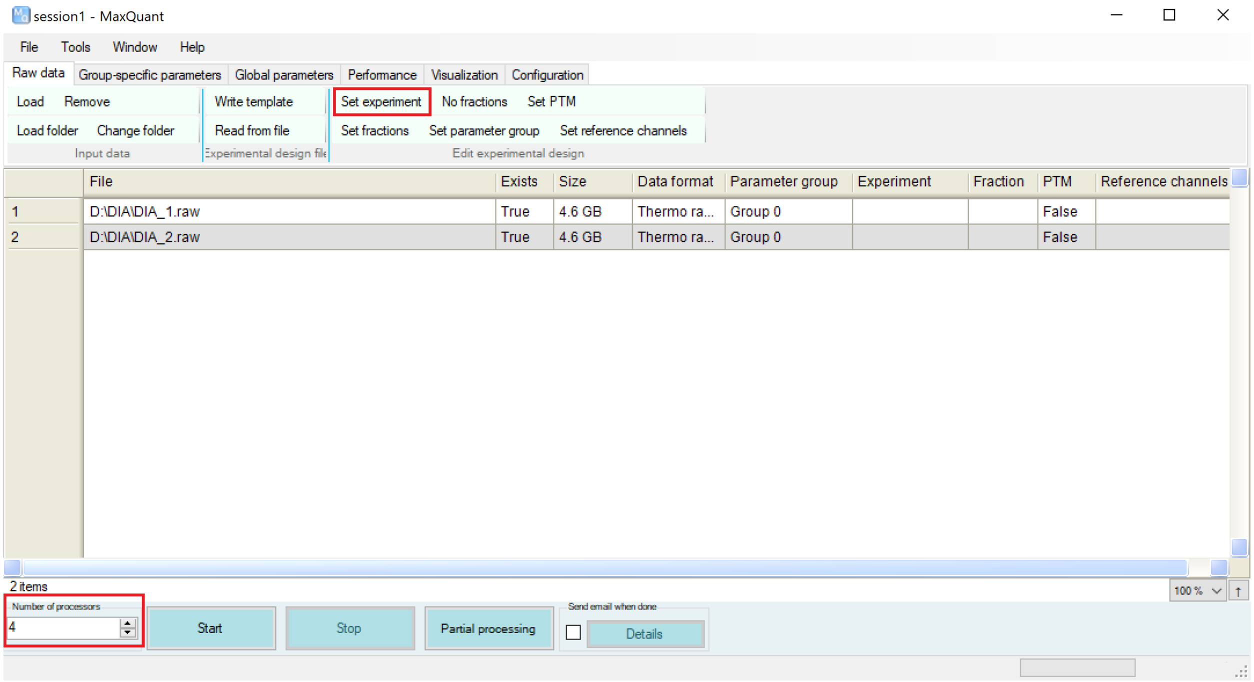

9. Now you can set the experiment design and the number of threads to be utilized by MaxQuant. Most PCs have two threads per core. You can simply press the Windows key on your PC and type “System Information”, press enter and look at the number of “Logical Processors” to find out the maximum number of threads you can set. It is

recommended to have at least 4 GB of Ram per utilized thread (e.g. 4 threads would need 16 GB of Ram).

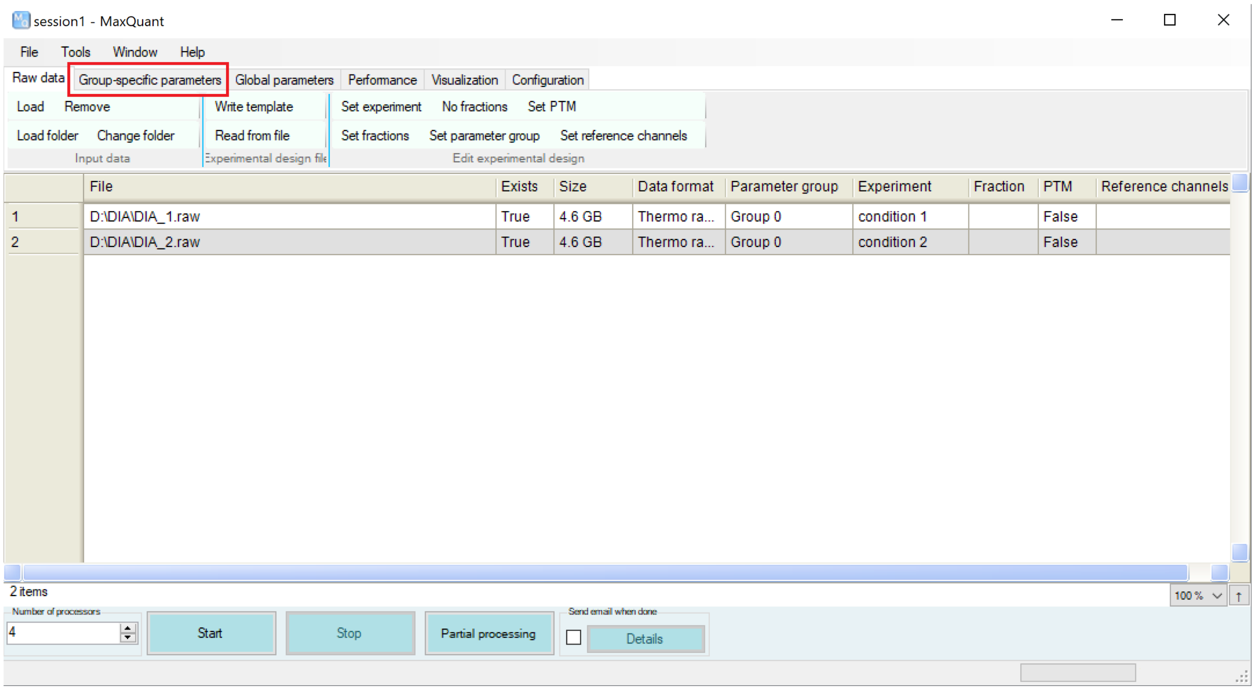

10. Next move on to the “Group-specific parameters” tab.

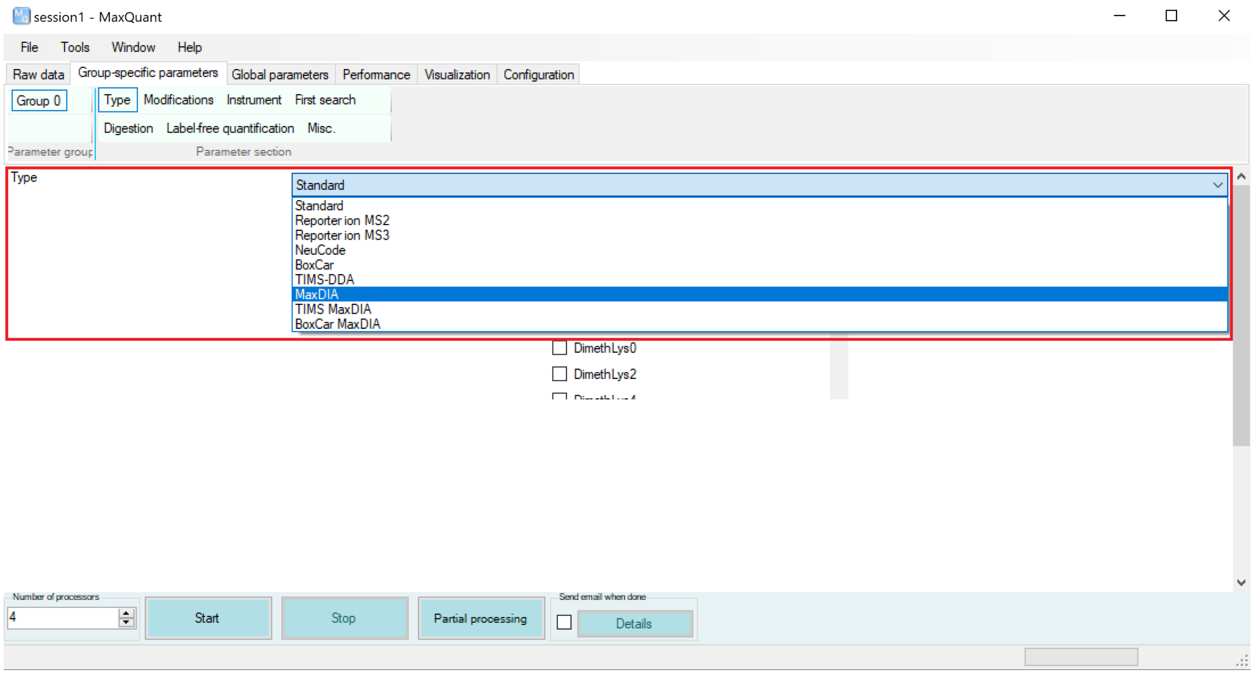

11. Here you can select the type of your mass spectrometry runs. There are three different MaxDIA algorithms available, MaxDIA, TIMS MaxDIA and BoxCar MaxDIA. Depending on your runs, choose the appropriate one.

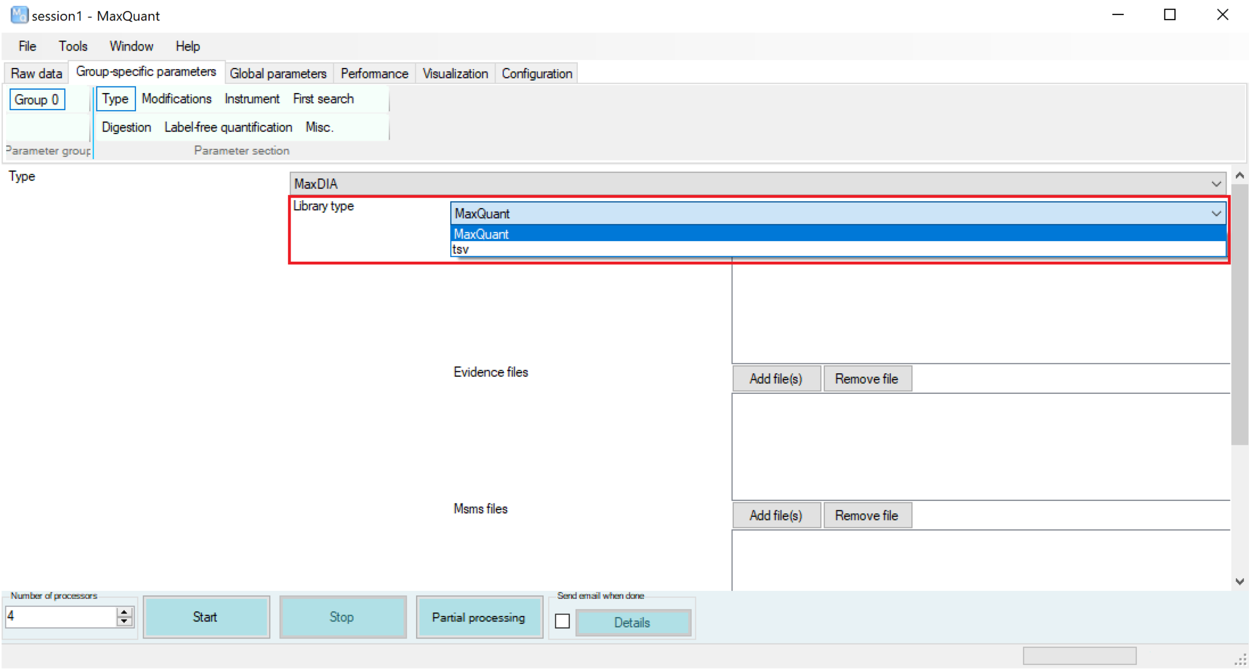

12. Next, you can choose the “Library type”. Choose “MaxQuant” for DDA library runs which have been processed with MaxQuant and “tsv” for other third party software which support a tsv output format.

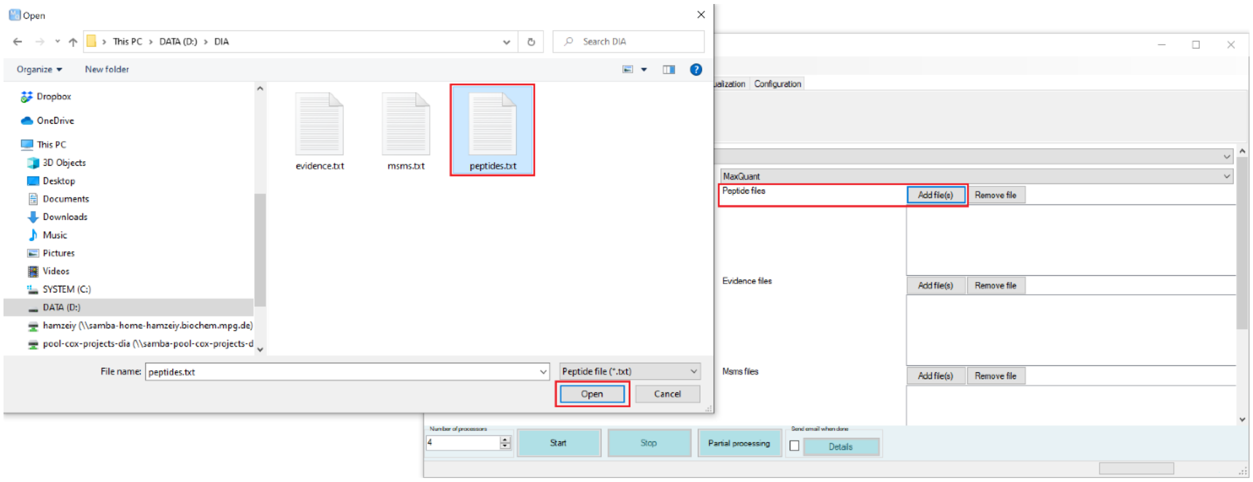

13. After choosing the library type, the library files should be added to each relevant section. The “peptides.txt”, “evidence.txt” and “msms.txt” files can be found in the “txt” folder of the “combined” folder of your DDA library runs with MaxQuant.

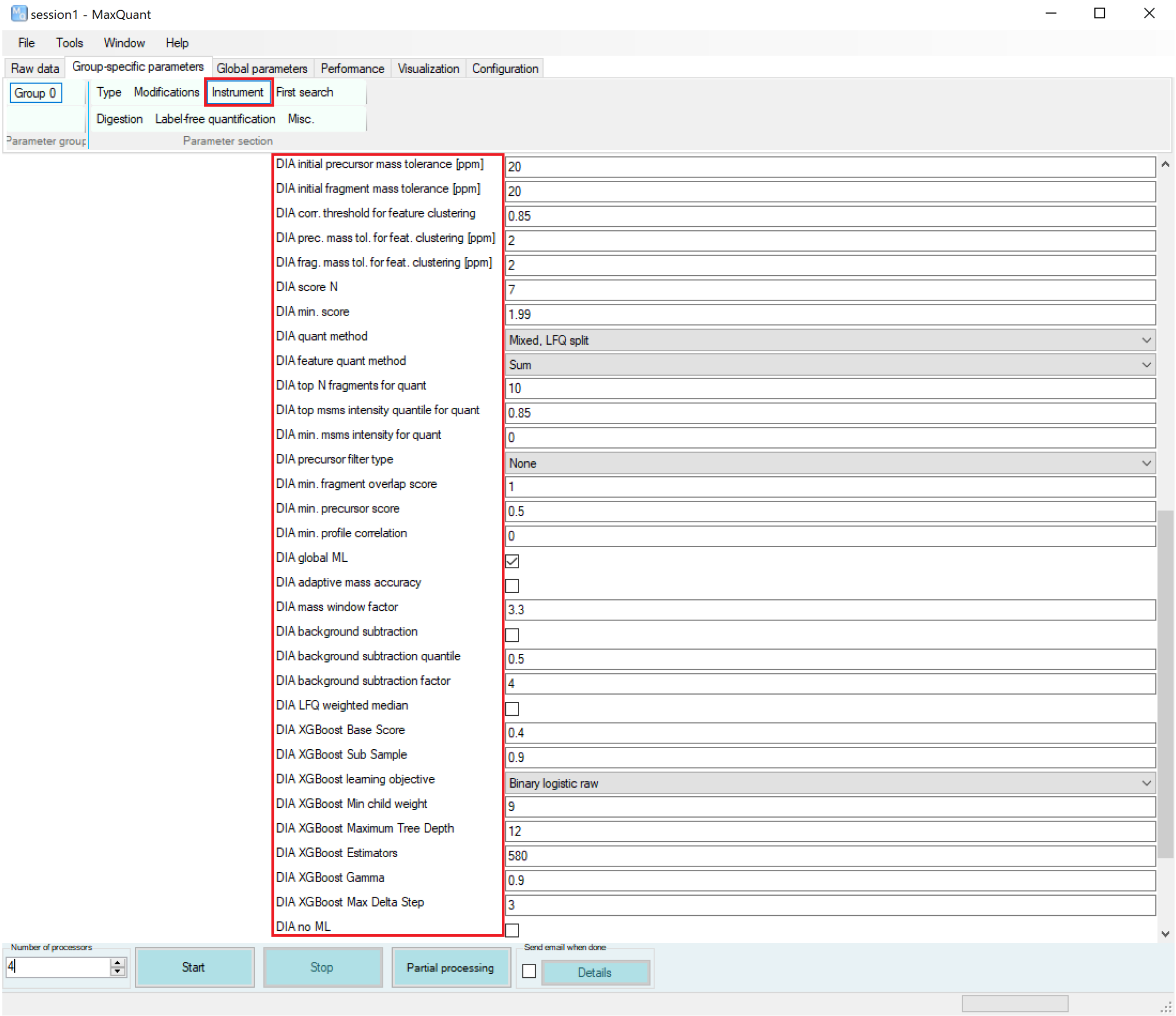

14. In the “Instrument” section, you can find many DIA related parameters. These parameters are further explained within the table at the end of this document.

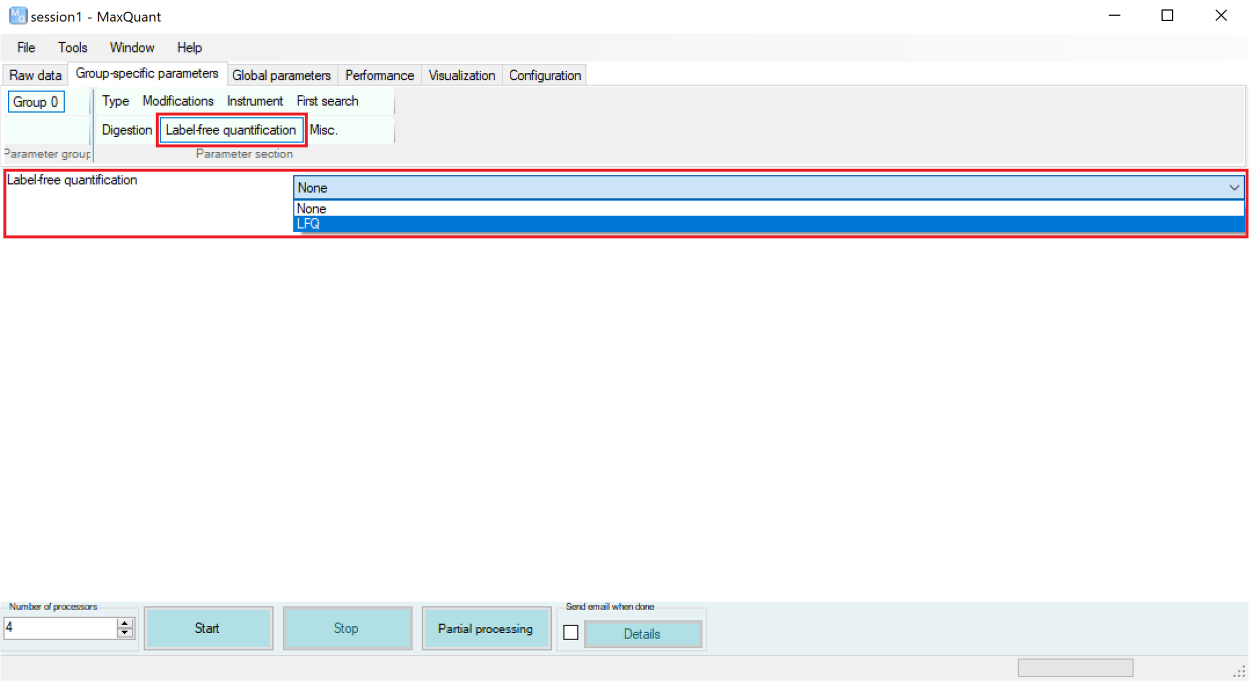

15. MaxQuant's label free quantification algorithm can be used for DIA samples too. To enable this, navigate to the "Label-free quantification" section and select "LFQ" from the drop-down menu.

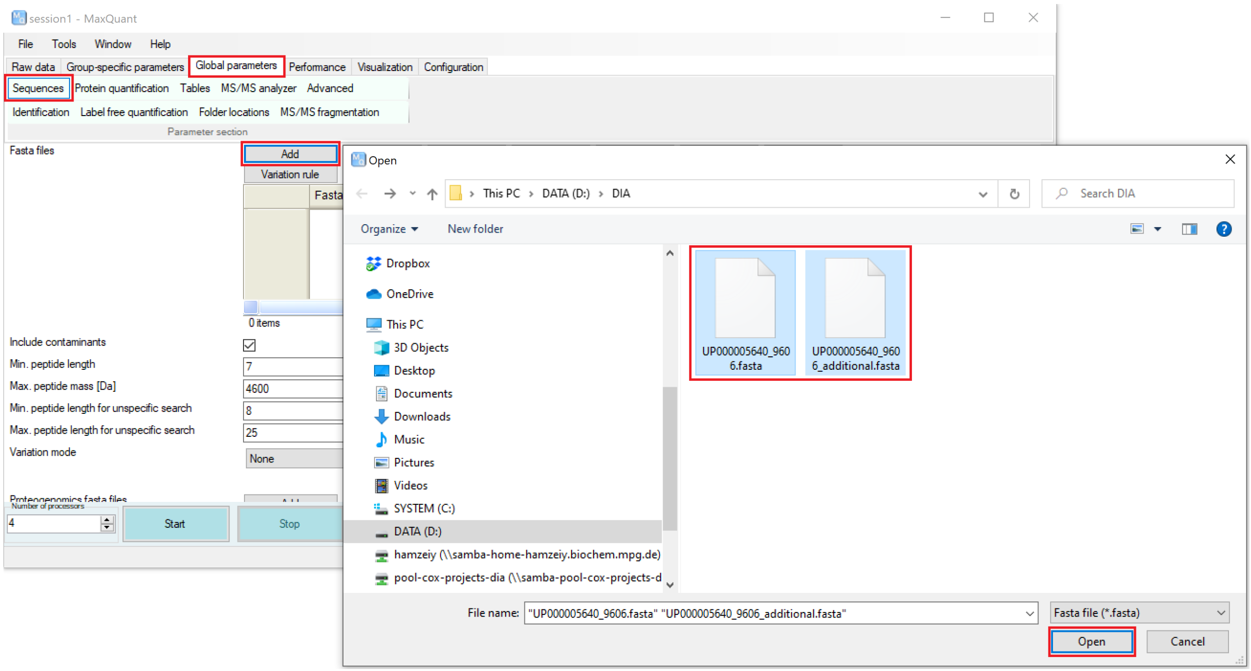

16. On the "Global parameters" tab, you can choose the appropriate FASTA files for your data under the "Sequences" section. You can download FASTA files for different organisms from the UniProt ftp server (ftp.uniprot.org) under: /pub/databases/uniprot/current_release/knowledgebase/reference_proteomes

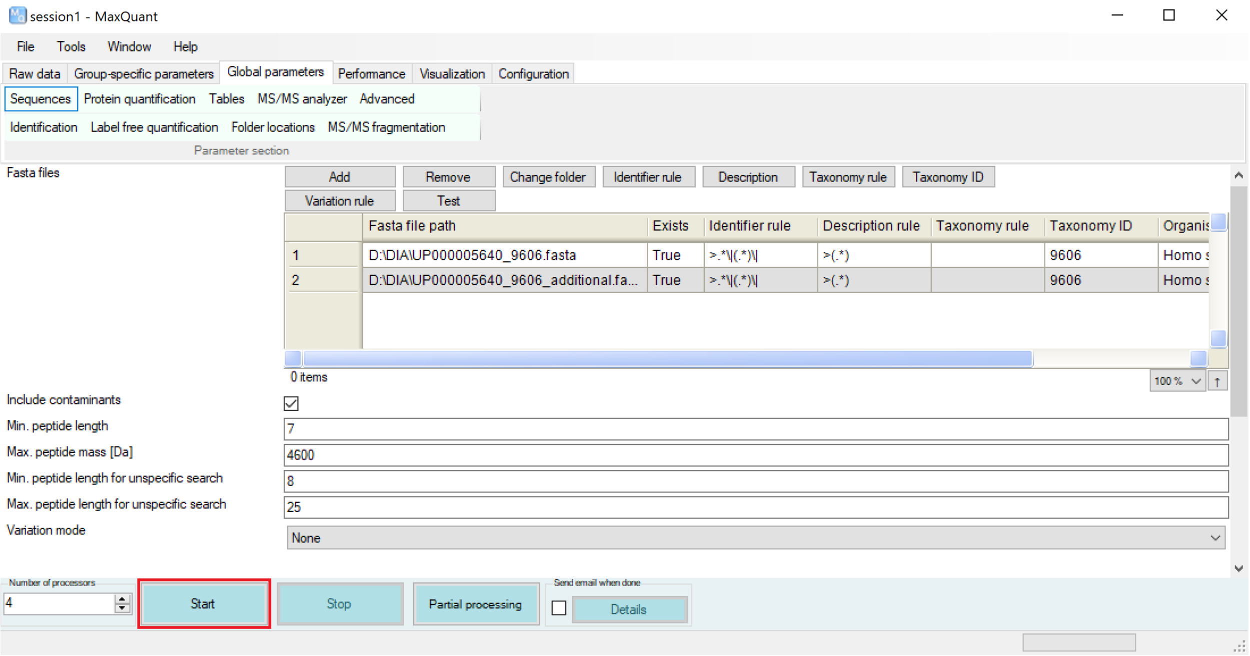

17. You can now start your analysis.

How to run MaxDIA in discovery mode

Summary: Running MaxDIA in discovery mode is identical to the library mode in every step except for the library files used (step 13 of library mode). Use *in silico* generated library files to run MaxDIA in discovery mode and the relevant FASTA files. Follow the steps below to download *in silico* libraries for most common species.

Steps:

1. Navigate to http://annotations.perseus-framework.org/.

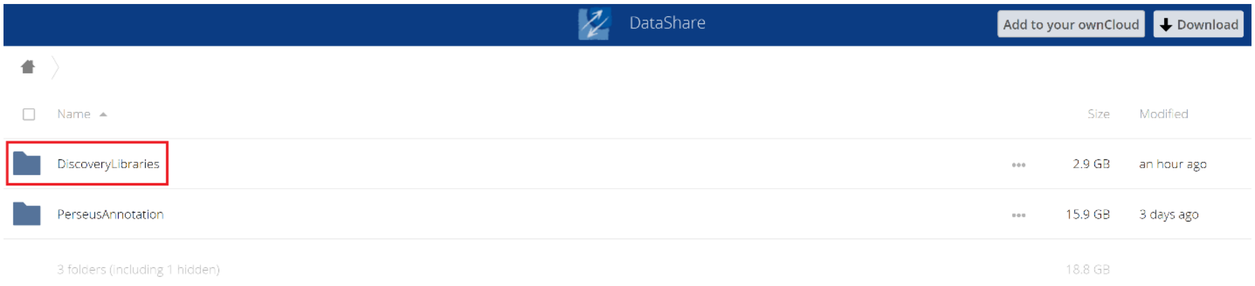

2. Click on "DiscoveryLibraries".

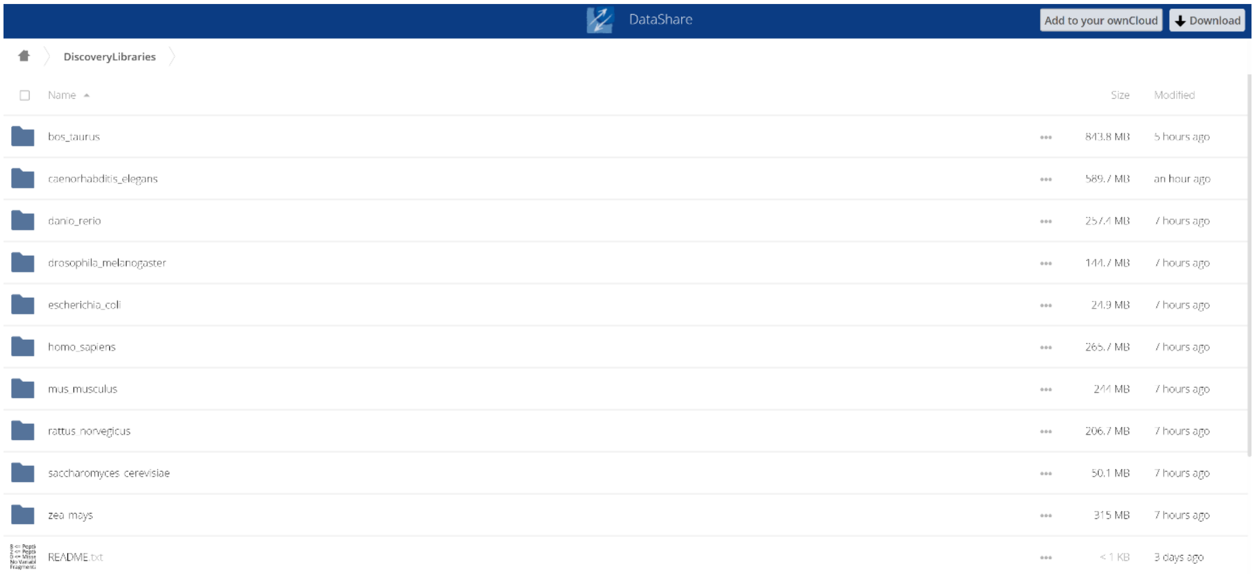

3. Here you can choose your organism of choice.

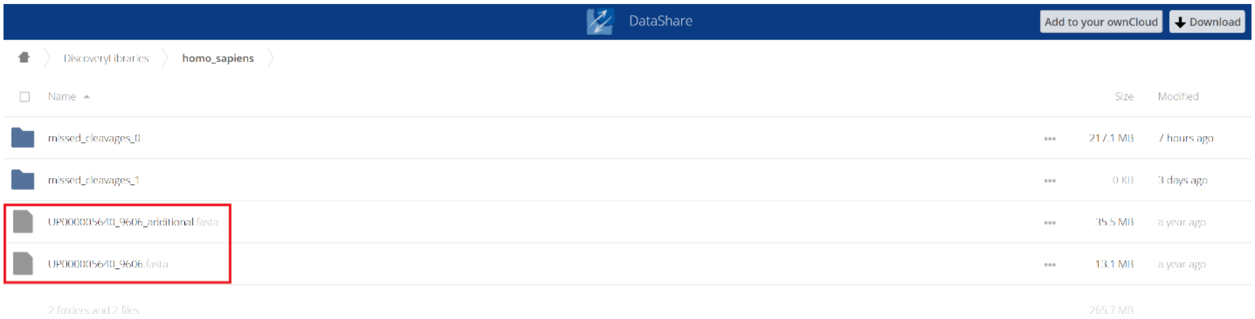

4. First download the relevant FASTA files. Then depending on the number of missed cleavages choose the relevant folder.

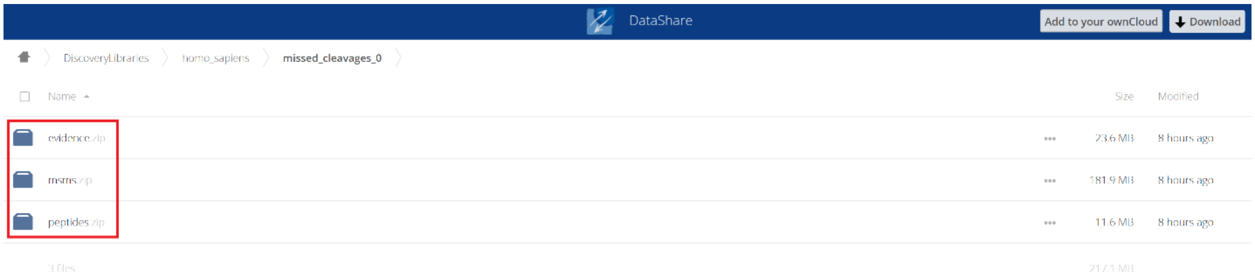

5. Here you can find the three library files needed for the discovery mode. You should unzip these files before use in MaxQuant.

How to submit results to the PRIDE repository

Summary: The PRIDE database has two main types of submissions “Complete Submission” and “Partial Submission”. The main different between both types of submissions is that in Complete Submissions the results (e.g. peptide and protein

evidences) are provided in a standard file format such as mzTab or mzIdentML. In addition, Complete submissions received a DOI. MaxQuant supports the mzTab file format to store its results, which is needed for the PRIDE complete submission. To generate the mzTab file, simply enable it from the “Tables” menu of the “Global parameters”.

Steps:

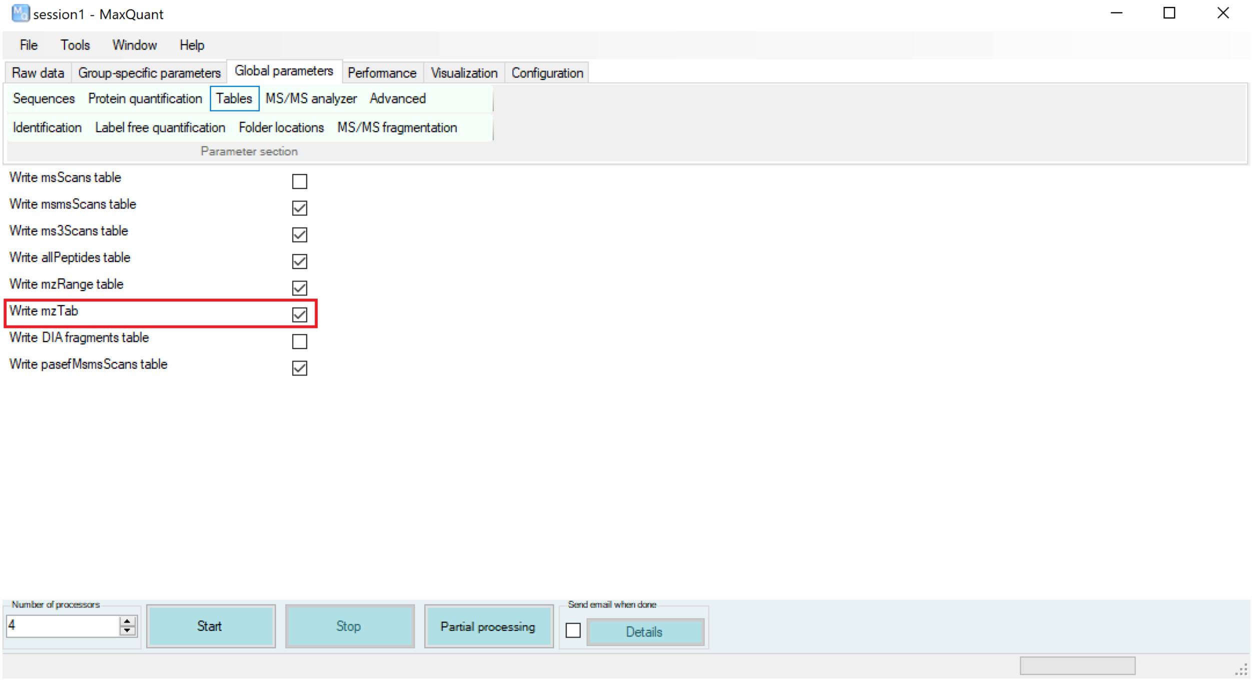

1. To enable the mzTab output file, simple enable it from the “Tables” menu of the “Global parameters”. It is disabled by default.

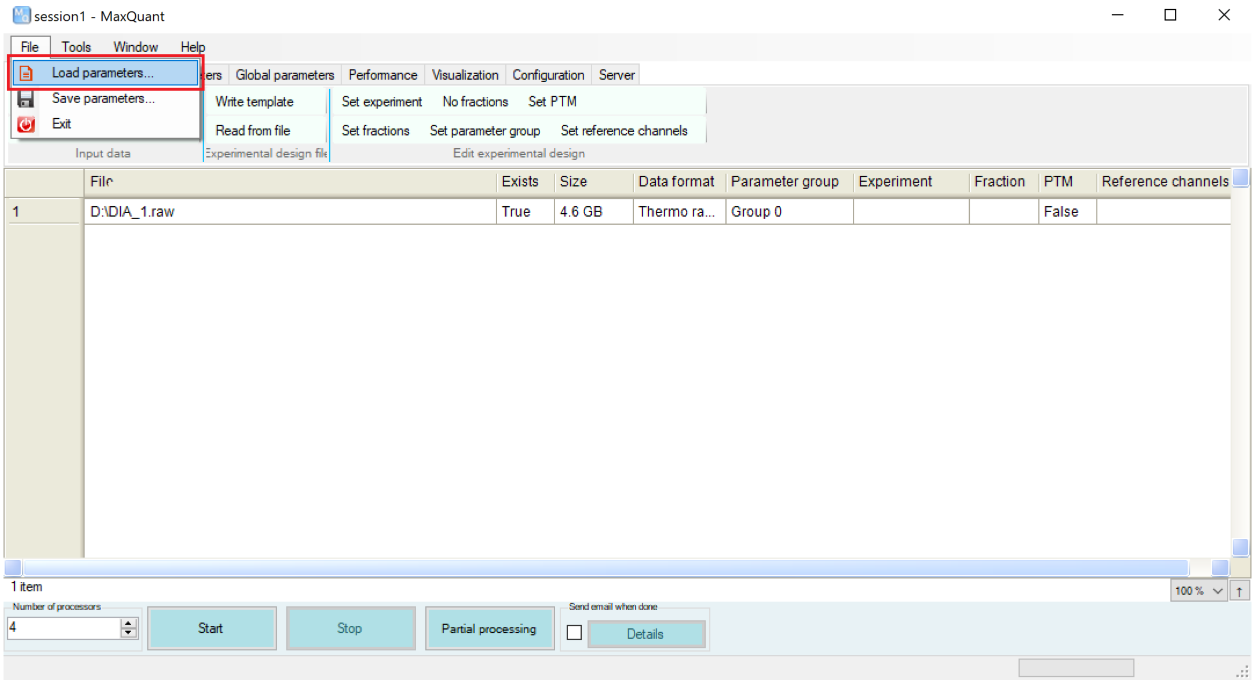

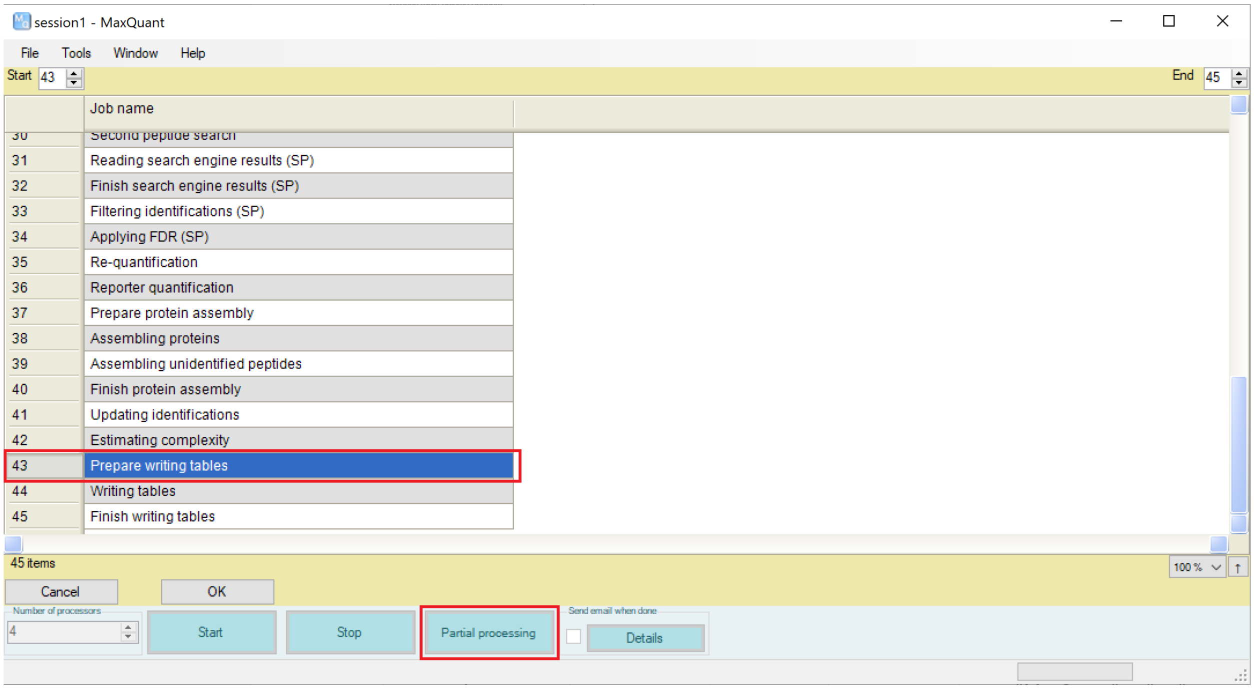

Note: You can also enable the mzTab option as described in step one and use "Partial processing" to simply only generate the mzTab file format for previously processed files by loading the relevant mqpar.xml file within the folder containing your raw mass spectrometry data.

Prepare the Pride Complete submission:

Summary: To make a complete Pride submission, you should download the submission tool from ProteomeXchange and follow the steps.

Steps:

1. Navigate to http://www.proteomexchange.org/submission/index.html.

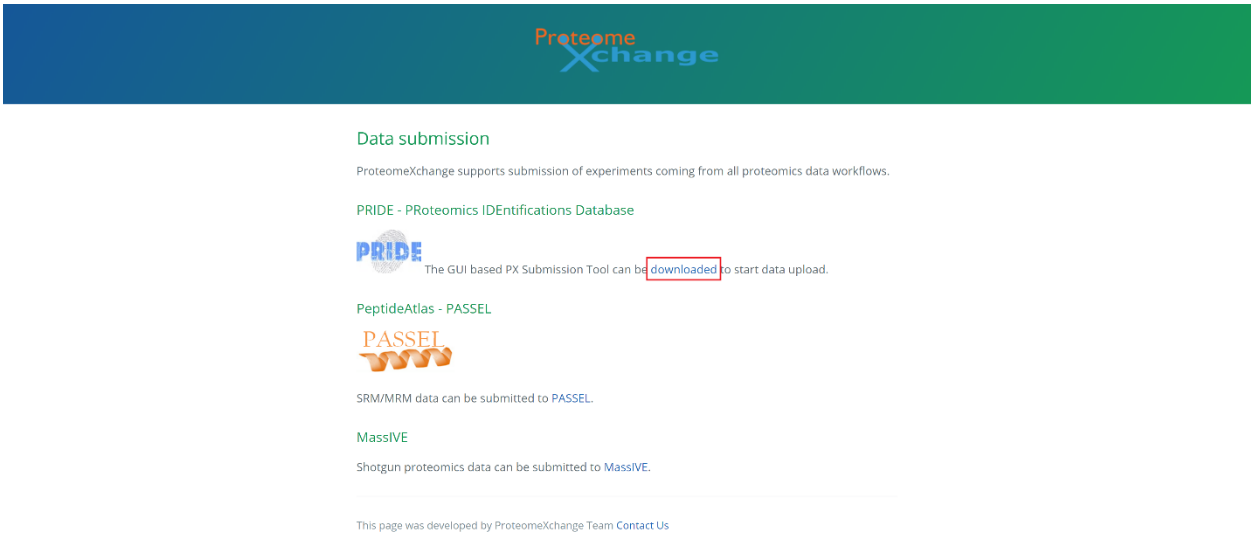

2. Download the submission tool and extract the contents of the zip file. Make sure to have java installed on your PC. The latest version of java can be downloaded and installed from https://www.java.com/en/download/.

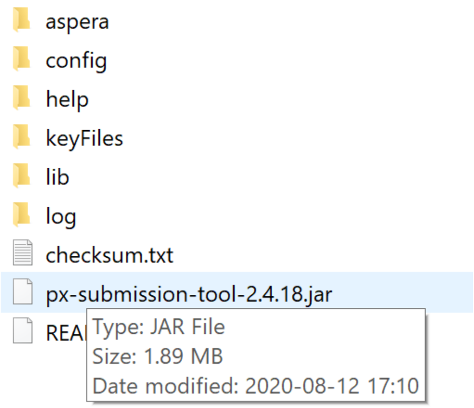

3. Double click on the jar file or refer to the README file for instruction on running the tool from the command line. Follow the steps accordingly.
4. After adding the title, sample and protocol description in the first two panels of the ProteomeXchange submission tool, the user will arrive to a panel where files should be provided:

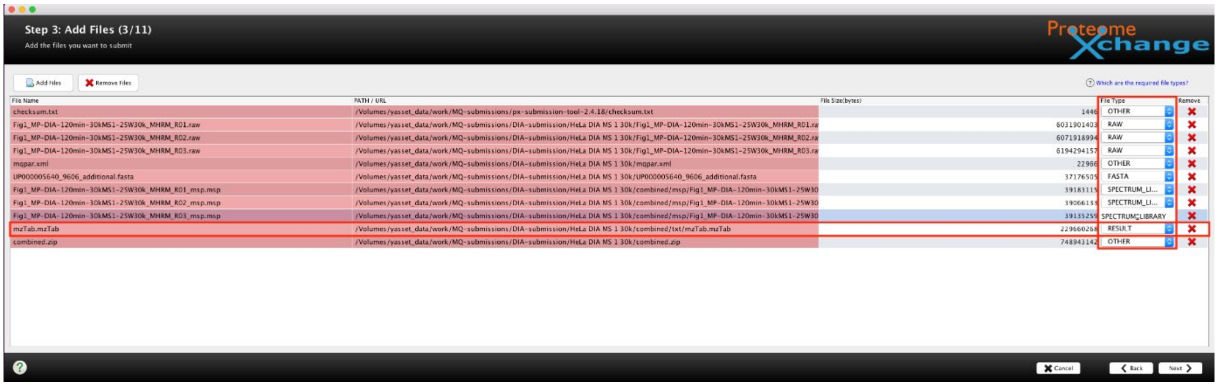

For MaxDIA Complete submissions the following files should be provided:

- **The mzTab File** (File Type RESULT): The mzTab contains the peptide and protein identifications in a standard file format including the references to the spectra use for the identification and the reference spectral library. The mzTab file is located in …/combined/txt/.
- **RAW files** (File Type RAW): The RAW files contain the original spectra capture by the mass spectrometer.
- **Protein FASTA database** (File Type FASTA): Protein database used in MaxDIA to map the peptides from the spectral library to the protein sequences.
- **Parameters file mqpar.xml** (File Type Other): The mqpar.xml contains all the parameters of the experiment including search parameters such as enzyme, modifications and statistical thresholds such FDRs. This file can be found where you have stored your RAW files.
- **Spectrum library references** (File Type Spectrum Library): MaxDIA generates with the mzTab a list of spectrum library files (extension MSP) which contains all the identified spectra from the original spectral library generated with the DDA data or the in-silico libraries. The MSP files are located in …/combined/msp/.
- **combined.zip** (File Type Other): In complete submissions it is important to provide also the MaxDIA combined folder in a compressed format. This folder contains additional information not included in the mzTab that are important for the users to understand the full experiment. This folder can be found where you have stored your RAW files.

Note: PRIDE recommends to perform two separate submissions for DDA and DIA data even if they are part of the same study. The user can cite or mention both accessions in the main manuscript. In this way, the DDA data used to generate the spectrum libraries can be submitted as one project and the DIA data with the resulting spectrum libraries from the DDA experiment can be submitted as a different project.

Table of all MaxDIA parameters

Parameter name (GUI)	Location in GUI Tabs	Location within GUI Tab	Parameter name (mqpar.xml)	Description
Type	Group-specific parameters	Type	lcmsRunType	This parameter can now be set to "MaxDIA", "TIMS MaxDIA" and "BoxCar MaxDIA" to turn on the MaxDIA algorithm for both library-based DIA and discovery DIA proccesing of LC-MS/MS-based proteomics runs.
Library type ("Type" must be set to "MaxDIA", "TIMS MaxDIA" or "BoxCar MaxDIA")	Group-specific parameters	Type	diaLibraryType	This parameter can be set to "MaxQuant" or "tsv", depending on the source of the library to be used for the MaxDIA algorithm
Peptide files ("Library type" must be set to "MaxQuant")	Group-specific parameters	Type	diaPeptidePaths	By clicking "Add file(s)", MaxQuant peptides.txt output file(s) or in silico peptides files in the MaxQuant output format can be defined
Evidence files ("Library type" must be set to "MaxQuant")	Group-specific parameters	Type	diaEvidencePaths	By clicking "Add file(s)", MaxQuant evidence.txt output file(s) or in silico evidence files in the MaxQuant output format can be defined
Msms files ("Library type" must be set to "MaxQuant")	Group-specific parameters	Type	diaMsmsPaths	By clicking "Add file(s)", MaxQuant msms.txt output file(s) or in silico msms files in the MaxQuant output format can be defined
Libraries ("Library type" must be set to "tsv")	Group-specific parameters	Type	diaLibraryPaths	By clicking "Add file(s)", library files in the tsv format can be defined
Min. DIA peak length	Group-specific parameters	Instrument	diaMinPeakLen	Minimum number of MS1 or MS2 scans for defining a 3D peak in DIA data
DIA initial precursor mass tolerance [ppm]	Group-specific parameters	Instrument	diaInitialPrecMassTolPpm	Indicates the mass tolerence for the initial search
DIA initial fragment mass tolerance [ppm]	Group-specific parameters	Instrument	diaInitialFragMassTolPpm	
DIA corr. threshold for feature clustering	Group-specific parameters	Instrument	diaCorrThresholdFeatureClustering	

DIA prec. mass tol. for feat. clustering [ppm]	Group-specific parameters	Instrument	diaPrecTolPpmFeatureClustering	
DIA frag. mass tol. for feat. clustering [ppm]	Group-specific parameters	Instrument	diaFragTolPpmFeatureClustering	
DIA score N	Group-specific parameters	Instrument	diaScoreN	
DIA min. score	Group-specific parameters	Instrument	diaMinScore	
DIA quant method	Group-specific parameters	Instrument	diaQuantMethod	Indicates the quantification method used for DIA data
DIA feature quant method	Group-specific parameters	Instrument	diaFeatureQuantMethod	
DIA top N fragments for quant	Group-specific parameters	Instrument	diaTopNForQuant	
DIA top msms intensity quantile for quant	Group-specific parameters	Instrument	diaTopMsmsIntensityQuantileForQuant	Indicates the top MS/MS intensity quantile to be used for quantification
DIA min. msms intensity for quant	Group-specific parameters	Instrument	diaMinMsmsIntensityForQuant	
DIA precursor filter type	Group-specific parameters	Instrument	diaPrecursorFilterType	
DIA min. fragment overlap score	Group-specific parameters	Instrument	diaMinFragmentOverlapScore	
DIA min. precursor score	Group-specific parameters	Instrument	diaMinPrecursorScore	
DIA min. profile correlation	Group-specific parameters	Instrument	diaMinProfileCorrelation	
DIA global ML	Group-specific parameters	Instrument	diaGlobalMl	Indicates whether to perform the machine learning on a per run basis or on the entire data set (global)
DIA adaptive mass accuracy	Group-specific parameters	Instrument	diaAdaptiveMassAccuracy	
DIA mass window factor	Group-specific parameters	Instrument	diaMassWindowFactor	
DIA XGBoost Base Score	Group-specific parameters	Instrument	diaXgBoostBaseScore	XGBoost base score parameter
DIA XGBoost Sub Sample	Group-specific parameters	Instrument	diaXgBoostSubSample	XGBoost sub sample parameter

DIA XGBoost learning objective	Group-specific parameters	Instrument	diaXgBoostLearningObjective	XGBoost learning objective parameter
DIA XGBoost Min child weight	Group-specific parameters	Instrument	diaXgBoostMinChildWeight	XGBoost minimum child weight parameter
DIA XGBoost Maximum Tree Depth	Group-specific parameters	Instrument	diaXgBoostMaximumTreeDepth	XGBoost maximum tree depth parameter
DIA XGBoost Estimators	Group-specific parameters	Instrument	diaXgBoostEstimators	XGBoost estimators parameter
DIA XGBoost Gamma	Group-specific parameters	Instrument	diaXgBoostGamma	XGBoost gamma parameter
DIA XGBoost Max Delta Step	Group-specific parameters	Instrument	diaXgBoostMaxDeltaStep	XGBoost maximum tree depth parameter
DIA no ML	Group-specific parameters	Instrument	diaNoMl	Parameter to turn off the machine learning
